# Combinatorial Synthesis of BaCu_2_Se_2_ Thin Films: Effect of Composition on Crystal Structure and Optoelectronic Properties

**DOI:** 10.1002/smll.73267

**Published:** 2026-04-02

**Authors:** Marin Rusu, José A. Márquez, Hannes Hempel, Galina Gurieva, Leo Choubrac, Ibrahim Simsek, Rene Schwiddessen, Pablo Reyes‐Figueroa, Daniel Molpeceres, Robert Wenisch, Markus Schleuning, Jan‐Ekkehard Hoffmann, Klaus Habicht, Christian A. Kaufmann, Iver Lauermann, Susan Schorr, Thomas Unold

**Affiliations:** ^1^ Department Structure and Dynamics of Energy Materials Helmholtz‐Zentrum Berlin für Materialien und Energie GmbH Berlin Germany; ^2^ Competence Centre Photovoltaics Berlin (PVcomB) Helmholtz‐Zentrum Berlin für Materialien und Energie GmbH Berlin Germany; ^3^ Institute Solar Fuels Helmholtz‐Zentrum Berlin für Materialien und Energie GmbH Berlin Germany; ^4^ Department Dynamics and Transport in Quantum Materials Helmholtz‐Zentrum Berlin für Materialien und Energie GmbH Berlin Germany; ^5^ Institut für Physik und Astronomie Universität Potsdam Potsdam Germany; ^6^ Institute of Geological Sciences Freie Universität Berlin Berlin Germany

**Keywords:** BaCu_2_Se_2_, bandgap, chalcogenides, combinatorial libraries, crystal structure, electronic properties, thin films

## Abstract

The BaCu_2_Se_2_ semiconductor compound with earth‐abundant elements is currently studied for application in thermoelectrics. The properties of this emerging material, however, are still not well established, while being mostly investigated on powders or pelletized powders. In this work, BaCu_2_Se_2_ thin films are prepared, and the chemical space of Ba‐rich and Cu‐rich compositions is explored. Ba‐Cu‐Se thin film combinatorial libraries with lateral compositional gradients with [Cu]/([Ba]+[Cu]) (CBC) atomic ratios between 0.15 and 0.98 are synthesized on areas of 12 × 50 mm^2^. Using XRF and XRD analysis, composition‐resolved phase fractions are determined, which identify the orthorhombic *α*‐BaCu_2_Se_2_ as the main phase for CBC between 0.62 and 0.82. Optical measurements reveal for *α*‐BaCu_2_Se_2_ a direct bandgap of 1.89 eV and a high absorption coefficient ∼10^5^ cm^−1^. Surface photovoltage and Seebeck coefficient measurements show *p*‐type conductivity of the films irrespective of composition. Combined Kelvin probe, photoelectron yield, and optical‐pump terahertz‐probe spectroscopies demonstrate tunability of charge carrier concentrations over several orders of magnitude. The absolute photoluminescence hyperspectral imaging reveals a bright luminescence centered at 1.91 eV and an implied open‐circuit voltage of 1.3 eV. Ba‐rich conditions are found to be appropriate for the synthesis of films for photovoltaics, while Cu‐rich conditions are suitable for the preparation of this material for thermoelectrics.

## Graphical abstract

The emerging BaCu_2_Se_2_ semiconductor compound is synthesized in combinatorial thin film libraries for fast screening of its fundamental properties related to the application in sustainable energy sources. Ba‐rich and Cu‐rich compositions are explored. Tunability in a large range of the material electronic properties makes BaCu_2_Se_2_ attractive for application in photovoltaics and thermoelectrics.

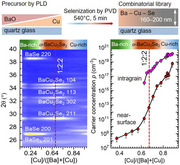

## 1 Introduction

The global demand for sustainable and green energy sources has prompted the development of photovoltaic (PV) devices due to their ability to convert sunlight directly into electricity. Among materials used as absorbers in the most efficient PV devices, multinary copper chalcogenides with the general formula $A^{I}B^{III}C_{2}^{VI}$ are of special interest since those show promising optoelectronic properties and can be deposited as thin films on large areas. In this class of materials, Cu(In,Ga)Se_2_ (CIGS) has shown excellent optoelectronic properties yielding laboratory‐scale thin film solar cells with efficiencies > 23% for single‐junction devices and >24% (≈1 cm^2^ active area), and > 27% (≈0.1 cm^2^ active area) in monolithic tandems with CIGS and halide perovskite thin films [1, 2, 3]. On the commercial scale, CIGS solar modules have demonstrated market maturity with stable efficiencies and long‐term operation, comparable to devices from crystalline silicon [4, 5]. However, the dependence on rare elements such as indium and gallium hampers their sustainable widespread application [6, 7]. To overcome this issue, various elemental substitution studies were performed to eliminate the toxic and scarce elements. It was proposed to replace the rare *B^III^* elements by zinc and tin, and extensive studies were carried out to establish a new quaternary absorber with Cu_2_ZnSn(S,Se)_4_ (CZTSSe) elemental constituents resulting in a kesterite‐type structure [8, 9]. Concentrated efforts during the last decades on achieving highly efficient CZTSSe solar cells have led to a maximum efficiency of ≈16%, though still significantly below that of CIGSe single‐junction cells [10]. This performance gap is primarily attributed to anti‐site defects with large capture cross‐sections for photogenerated carriers [11, 12]. Comprehensive studies of physicochemical and thermodynamic limits by employing photovoltaic analysis of different Zn substitutes (Mn, Mg, Fe, Ni, Co, Ba, Sr) in CZTSSe have revealed Ba (II) as a potential element to replace Zn [13, 14]. The latter replacement resulted in an earth‐abundant and environment‐friendly material Cu_2_BaSn(S,Se)_4_ (CBTSSe). Being yet an emerging absorber material, solar cells based on CBTSSe with an efficiency of 6.5% were recently demonstrated, and the potential of reaching efficiencies > 20% was shown [15, 16]. However, due to the multinary composition, a precise control of processing parameters for the deposition of both CBTSSe and CZTSSe films is needed. In addition, the deposition of single‐phase films is challenging due to a narrow phase stability range, which implies a high probability of incorporating secondary phases [8, 11]. Considering the beneficial effects of Ba in the latter quaternary films and the further reduction of the number of elements involved in the material composition, we choose to investigate the emerging BaCu_2_Se_2_ semiconductor compound for fast screening of its physical properties and potential application in sustainable energy sources by primarily focusing on PV. BaCu_2_Se_2_ is an earth‐abundant and environmentally benign BaCu_2_Se_2_ chalcogenide semiconductor compound composed of divalent Ba^2+^ cations, monovalent transition metal Cu^+^ cations, and divalent Se^2−^ anions. Despite these beneficial properties, up to now there have been no studies reported on the deposition of thin films, while only a few papers can be found on the preparation of BaCu_2_Se_2_ in the form of single crystals [17, 18] and powders or pelletized powders [19, 20, 21, 22, 23]. Also, only a few works were reported on theoretical studies based on first‐principles density functional theory (DFT) calculations [19, 24, 25, 26, 27]. The crystal structure of BaCu_2_Se_2_ was studied by a few groups to date [17, 18, 19, 20, 22]. Two polymorphs were reported: (i) *α*‐BaCu_2_Se_2_ with an orthorhombic crystal structure (space group *Pnma*) and (ii) *β*‐BaCu_2_Se_2_ with the tetragonal structure of ThCr_2_Si_2_ (space group *I*4/*mmm*) [18]. Experimental and computational results indicate a stability of orthorhombic *α*‐BaCu_2_Se_2_ polymorph up to temperatures of 540 °C and 650 °C, respectively [18, 22]. An irreversible phase transition from the orthorhombic phase to the tetragonal phase is reported to occur at temperatures > 540 °C [18] via a first‐order reconstructive mechanism [22]. Calculated band structures yielded quasi‐direct or direct bandgaps between 1.30 and 1.70 eV for the *α*‐phase [19, 23, 24, 25, 26, 27] while a lower band gap of 0.98–1.38 eV was determined for the *β*‐phase [25, 27]. First experimental band gap values determined from UV–vis measurements vary between 1.63 and 1.8 eV [19, 20, 23]. The absorption coefficient is reported only for computed data, yielding values of ≈5 × 10^4^ cm^−1^ for photon energies above the onset of strong absorption calculated at ∼2 eV [19]. BaCu_2_Se_2_ has been reported to be of *p*‐type conductivity with hole concentrations between 1.7 and 3.2 × 10^18^ cm^−3^ and mobilities between 10 and 18 cm^2^ V^−1^ s^−1^ [19, 20, 23, 27]. The high concentration of holes in pristine samples is explained to originate from Cu vacancy (*V*_Cu_) defects. Higher hole concentrations of 2.8 × 10^19^ cm^−3^ were obtained by *V*_Cu_ engineering [23]. A further increase of the carrier concentration to 2.6 × 10^20^ cm^−3^ was attained by substituting Ba^2+^ by Na^+^ [20]. High charge carrier concentrations along with the reported low thermal conductivity fit well to the requirements for obtaining high‐performance thermoelectric (TE) materials, i.e., materials characterized by a high figure of merit ZT = *σ S* ^2^ *T*/*κ*_t_, where *T* is the operating temperature, and *σ*, *S*, and *κ*_t_ are the electrical conductivity, Seebeck coefficient, and thermal conductivity, respectively [28]. That is why BaCu_2_Se_2_ is investigated to date primarily for TE applications. However, very little work has been reported on the fundamental properties of this material in relation to its application in photovoltaic systems. In addition, the available publications still show very large deviations between computed and experimentally determined bandgaps, e.g., 1.3 eV computed vs. 1.8–1.9 eV measured. While high carrier concentrations of about 10^19^ cm^−3^ were reported on synthesized polycrystalline materials, no data is available on the possibility of decreasing and controlling those values. Also, no studies reported the preparation of thin films. It is therefore the main goal of this work to synthesize BaCu_2_Se_2_ thin films and investigate thoroughly their structural, electrical, and optoelectronic properties as a function of composition. We present the exploration of the chemical space of combinatorial BaCu_2_Se_2_ thin films with a wedge gradient in their [Cu]/([Ba] + [Cu]) ≡ CBC atomic ratios, which were synthesized on quartz glass substrates (12 × 50 mm^2^) by selenization of Cu‐BaO precursors from pulsed laser deposition (PLD). The elemental composition of these films was mapped by using an X‐ray fluorescence (XRF) analysis, and the thin film thickness distribution was calculated. The structural properties were investigated by grazing incidence X‐ray diffraction (GIXRD). The optoelectronic properties of the films were assessed by high‐throughput, contactless methods. To determine the optical bandgaps and absorption coefficients as a function of thin film composition, transmission and diffuse reflectance spectra were collected. Further, photoluminescence (PL), surface electronic properties, transport, and the thermoelectric power factor were investigated.

## 2 Results and Discussion

### 2.1 Compositional Gradients, Crystal Structure, and Development of Phases

**FIGURE 1:**
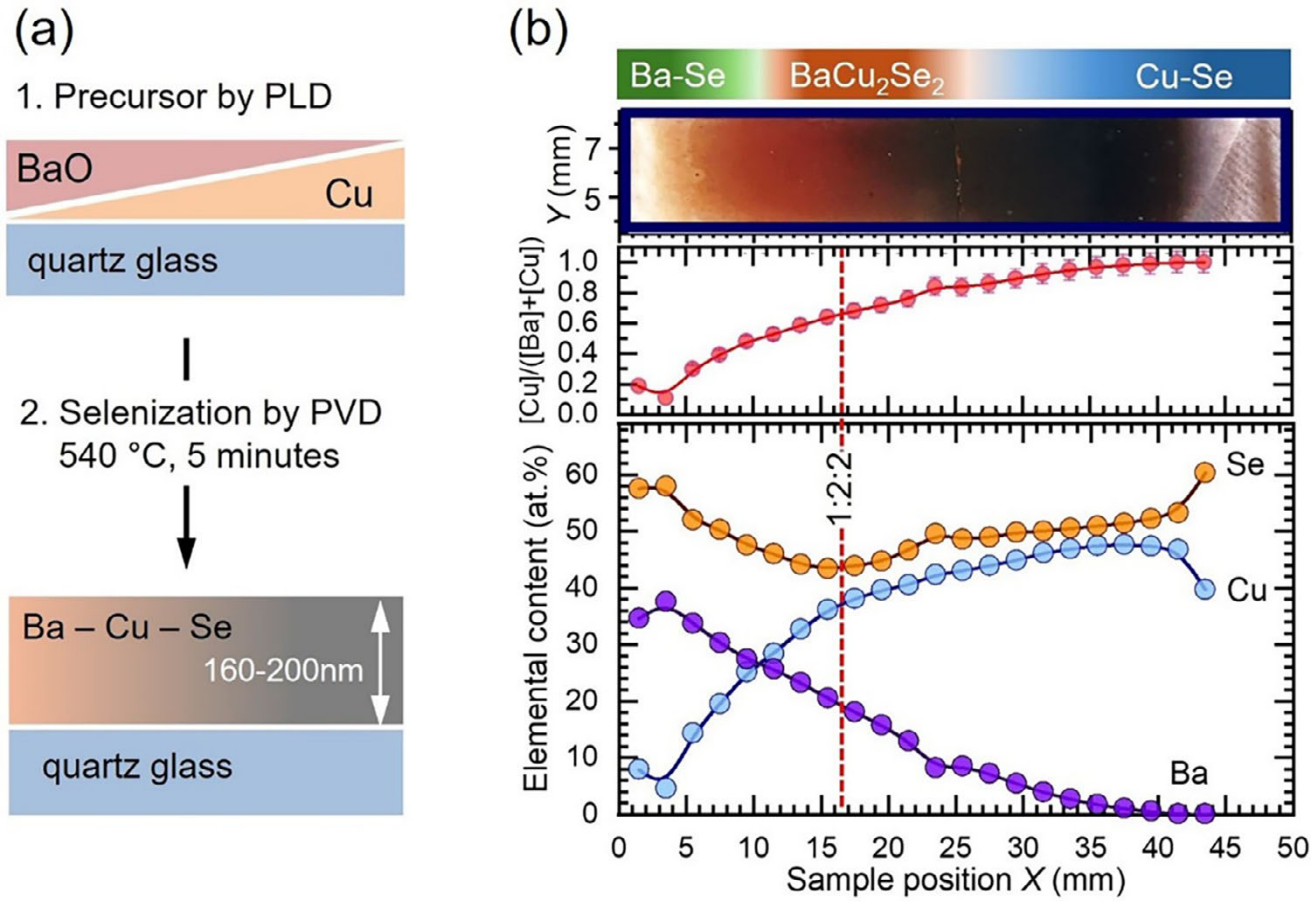
(a) Schematic of the preparation stages of the combinatorial Ba‐Cu‐Se thin films on quartz glass substrates. In the first preparation stage, precursor films consisting of BaO and Cu thin film wedges were prepared by pulsed laser deposition (PLD) at room temperature. In the second stage, the PLD‐precursors were selenized in a physical vapor deposition (PVD) system at 540 °C. (b) (top) Optical image of the Ba‐Cu‐Se combinatorial film with a color bar indicating regions of expected phases. (middle) [Cu]/([Ba]+[Cu]) atomic ratio as a function of lateral position (*X*) obtained from X‐ray fluorescence (XRF) analysis. (bottom) Elemental composition profiles of Ba, Cu, and Se along the same direction derived from XRF data.

**FIGURE 2:**
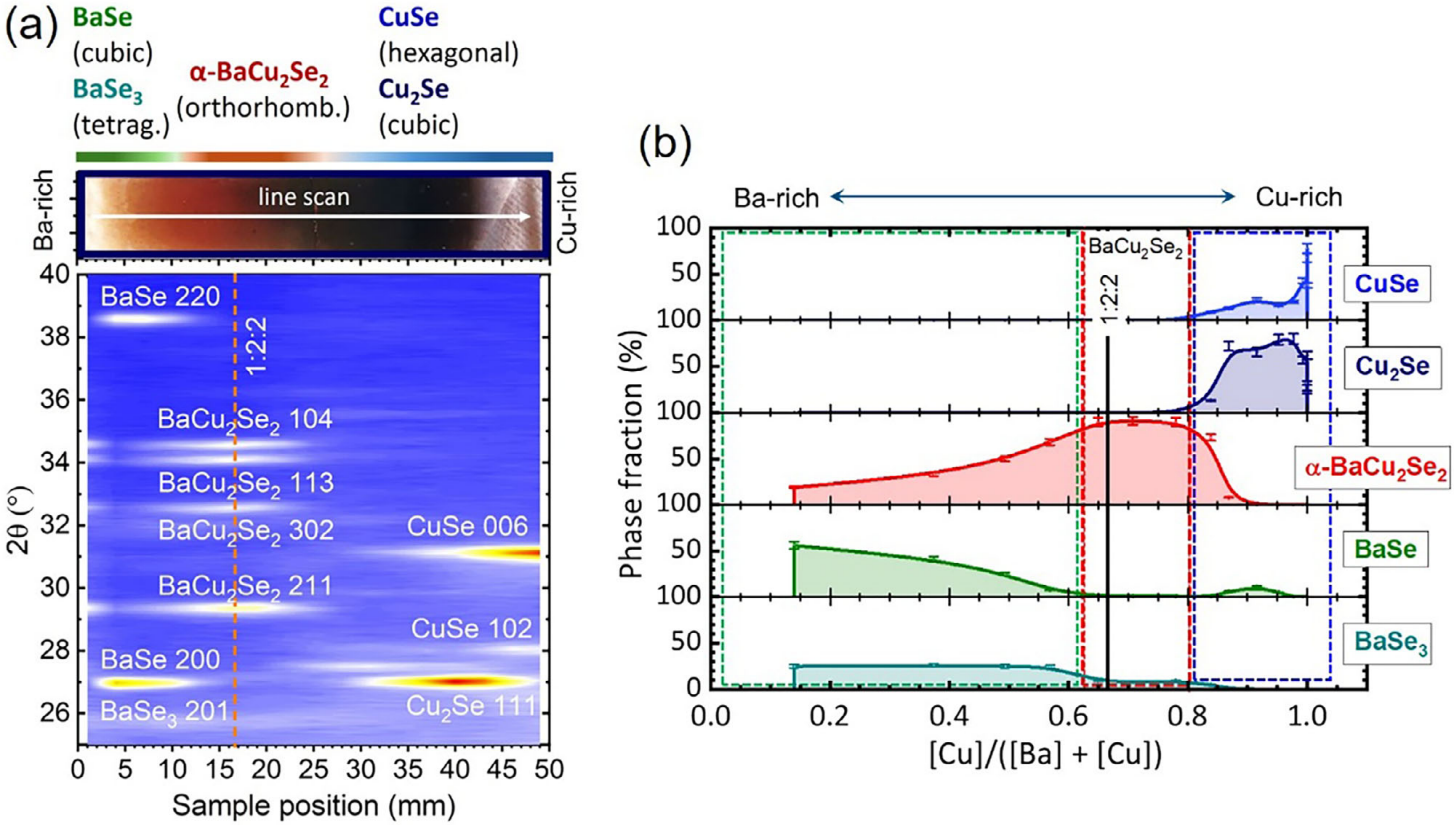
(a) Grazing incidence X‐ray diffraction (GIXRD) patterns from the line‐scan of the combinatorial sample as indicated on the optical image. The obtained GIXRD map visualizes the phase evolution and the interpenetrating regions of different phases as a function of position on the sample. Miller indices hkl were assigned by comparison with reference peak positions of the cubic BaSe (space group $Fm\bar{3}m$) [30], tetragonal BaSe_3_ (space group $P\bar{4}2_{1}m$) [31], orthorhombic α‐BaCu_2_Se_2_ (space group *Pnma*) [22], hexagonal klockmannite CuSe (space group *P6_3_/mmc*) [32], and cubic Cu_2_Se (space group *Fm3m*) [33], and performing Le Bail refinements. (b) Phase fractions as a function of composition, as calculated from the GIXRD line‐scan and the assignment of corresponding [Cu]/([Ba]+[Cu]) ratios to sample positions as determined from XRF measurements in Figure 1b.

To evaluate the influence of the elemental composition on BaCu_2_Se_2_ material physical properties, combinatorial Ba‐Cu‐Se thin films were synthesized on quartz glass substrates using a two‐stage preparation procedure, which is schematically illustrated in Figure 1a. In the first stage, the BaO and Cu wedge films were prepared by PLD at room temperature. In the second stage, the wedge precursors were selenized at a substrate temperature of 540 °C for 5 min, resulting in the formation of the Ba‐Cu‐Se combinatorial gradient along the thin film with the thickness varying between 160 and 200 nm (see Experimental Section for details). Figure 1b shows the optical image of the as‐synthesized sample, which reveals clear changes in color related to variations of optical properties due to changes in the chemical composition of the thin film. The chemical composition of the sample was mapped by using XRF analysis under consideration that (i) the selenization is complete and (ii) the amount of the remanent oxygen in the film can be neglected since it shall be eliminated from the system as bound in the easily volatile SeO_2_ with a low sublimation temperature of 317°C, which forms as a result of reaction between BaO and Se as 2BaO + 3Se → 2BaSe + SeO_2_ [29]. The loss of oxygen was verified by energy dispersive X‐ray spectroscopy (EDS). Within the EDS detection limits, no oxygen was detected (see Figure S1). We note, however, that traces of oxides such as BaO_2_, CuO, Cu_2_O, and hydroxides such as Ba(OH)_2_ and Cu(OH)_2_ were found by X‐ray photoelectron spectroscopy on the surface of the air‐exposed samples. Figure 1b (bottom panel) shows the XRF elemental concentration profiles, which demonstrate the realization of the Ba and Cu gradients. A monotonic increase of the relative CBC atomic ratio is observed (Figure 1b, middle panel) moving from the Ba‐rich light red to the brown‐red and further to the Cu‐rich black regions of the sample. A variation of the CBC atomic ratio of about 80% along the 50 mm sample is observed. The chemical composition corresponding to the CBC = 0.67, which matches the stoichiometric 1:2:2 composition, is clearly resolved in an area slightly shifted from the middle of the sample. The almost absent Cu at the Ba‐rich edge of the sample suggests formation of Ba‐Se binary compounds, while the absence on Ba at the Cu‐rich sample edge points to the formation of Cu‐Se binaries. Indeed, the GIXRD mapping performed along the line‐scan in Figure 2a (see more details in Figure S2a, b) reveals at the Ba‐rich edge formation of the cubic BaSe (space group $Fm\bar{3}m$) [30] and tetragonal BaSe_3_ (space group $P\bar{4}2_{1}m$) [31]. At the Cu‐rich edge, GIXRD detects the hexagonal klockmannite‐type structure CuSe (space group *P6_3_/mmc*) [32] and cubic Cu_2_Se (space group *Fm3m*) [33]. At sample positions between ≈12 and ≈25 mm, which correspond to variations of the CBC atomic ratio between 0.57 and 0.78 in Figure 1b, i.e., around the 1:2:2 stoichiometry region, the identified Bragg peaks indicate the formation of the stable orthorhombic *α*‐phase BaCu_2_Se_2_ (space group *Pnma*) [22]. The Le‐Bail method [34] was applied for refinement of the diffraction patterns of the thin films with the stoichiometry close to 1:2:2. The refinement was performed by using the Full‐Prof Suite software package [35]. The data were well characterized by considering a model which included *α*‐BaCu_2_Se_2_ (*Pnma*), BaSe ($Fm\bar{3}m$) and BaSe_3_ ($P\bar{4}2_{1}m$). The analysis resulted in unit cell constants for the *α*‐BaCu_2_Se_2_ bulk of *a* = 9.594 (2) Å, *b* = 4.214 (1) Å and *c* = 10.771 (2) Å (see Figure S2c and Table S1) which are in very good agreement with the literature data published on pelletized powder [22]. Only a slight increase of the unit cell parameters is observed for the surface layer measured at an incident angle of 0.3°, which amounts to *a* = 9.599 (2) Å, *b* = 4.216 (1) Å, and *c* = 10.773 (2) Å. The GIXRD map indicates that there are regions which simultaneously contain multiple crystallographic phases. To elucidate the evolution of the phase contents as a function of elemental content, we have determined the phase fractions by fitting the XRD patterns, obtained for various CBC ratios, with the Powder Cell software [36]. The fittings were performed under the assumption of a uniform distribution of phases in the film depth. The results are plotted in Figure 2b. It can be seen that the Ba‐rich area contains the basic Ba‐Se phases, e.g., BaSe and BaSe_3,_ with an *α*‐BaCu_2_Se_2_ phase content of about 15 wt.%. The amount of the *α*‐BaCu_2_Se_2_ phase increases monotonically with the Cu content reaching the maximum of ≈93 wt.% close to the 1:2:2 stoichiometry (CBC ≈ 0.67). Despite further increase of the Cu content to CBC ≈ 0.75, the amount of the *α*‐BaCu_2_Se_2_ phase remains constant along with the content of the BaSe_3_ phase, while no Cu‐Se secondary phases are observed. The onset of the Cu‐Se phases is detected for CBC > 0.80. Starting with this atomic ratio, the *α*‐BaCu_2_Se_2_ phase vanishes quite abruptly. At the same composition, the amount of CuSe and especially of Cu_2_Se increases sharply. Elemental concentrations calculated by considering the corresponding phase fractions were found in good agreement with the data obtained from XRF analysis (see Figure S2d).

The morphology of the BaCu_2_Se_2_ thin films was probed by means of scanning electron microscopy (SEM). The SEM image from the combinatorial sample area with a composition around the 1:2:2 stoichiometry of BaCu_2_Se_2_ depicts parallelepiped‐shaped large grains with sizes between 50 and 400 nm, which are partially covered by small grains of about tens of nanometres (Figure S3a). The geometry of the observed parallelepiped‐shaped grains with orthogonal facets is in perfect agreement with the orthorhombic crystal system of the *α*‐BaCu_2_Se_2_ found from the XRD measurements. Since on areas with the stoichiometry close to 1:2:2 only the BaSe_3_ secondary phase was found, we attribute the small grains to the latter secondary phase. The segregation of the BaSe_3_ secondary phase on top of the films makes its removal easier by post‐deposition treatments (PDT), e.g., chemical surface treatments or thermal annealing. We have deposited *α*‐BaCu_2_Se_2_ films on Mo‐coated soda lime glass substrates with sizes of 12 × 15 mm^2^ and applied a thermal PDT at 530 °C for 30 min under N_2_ flux (5N purity) at a pressure of 100 mbar. This resulted in BaCu_2_Se_2_ films with a structure of layered terraces, which are composed of ordered parallelepiped‐shaped grains (see Figure S3b).

### 2.2 Optical Properties

#### 2.2.1 Absorption Coefficient and Bandgap

**FIGURE 3:**
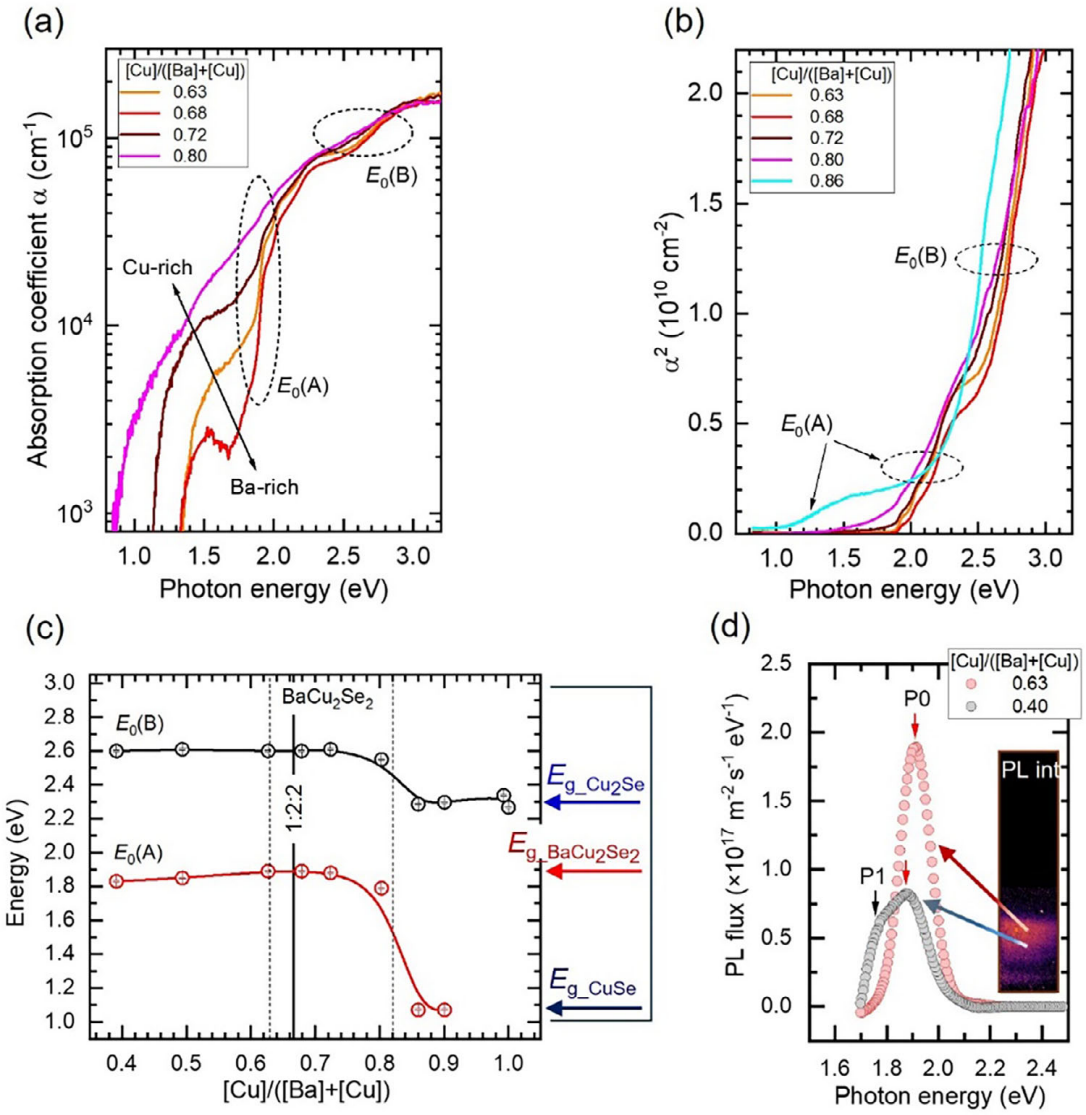
(a) Absorption coefficient spectra of BaCu_2_Se_2_ with different compositions around the 1:2:2 stoichiometry corresponding to a ratio [Cu]/([Ba]+[Cu]) = 0.67. Distinct *E*_0_(A) and *E*_0_(B) optical transitions are observed at about 1.9 and 2.6 eV, respectively. (b) Squared absorption coefficient spectra of Ba‐Cu‐Se combinatorial samples for the determination of the *E*_0_(A) and *E*_0_(B) energy values as a function of composition, which varies from Ba‐rich to Cu‐rich elemental contents. (c) *E*_0_(A) and *E*_0_(B) energy values of the Ba‐Cu‐Se combinatorial film as a function of the [Cu]/([Ba]+[Cu]) ratio. The energies of those optical transitions are found in very good agreement with the optical bandgap energies (*E*_g_) of materials detected by the GIXRD line‐scan as a function of composition in Figure 2a. (d) Photoluminescence (PL) yield of the Ba‐Cu‐Se film from the area of Ba‐rich compositions. The inset shows the PL intensity image of the entire combinatorial film.

To determine the optical bandgaps and the absorption coefficients as a function of thin film composition, transmission (*T*) and diffuse reflectance (*R*) spectra were collected from films deposited on quartz glass substrates (see Figure S4). Because of unknown optical properties and low film thicknesses *d*, between 160 and 200 nm, the absorption coefficient (*α*) as a function of CBC atomic ratio was calculated by applying the established formula $\alpha =\left(-1/d\right)\ln\left[\left(\sqrt{{\left(1-R\right)}^{4}+4T^{2}R^{2}}-{\left(1-R\right)}^{2}\right)/2TR^{2}\right]$ [37, 38]. Selected absorption coefficient spectra for BaCu_2_Se_2_ compositions around 1:2:2 stoichiometry indicate two distinct optical transitions *E*_0_(A) and *E*_0_(B) at energies of about 1.9 and 2.6 eV, respectively (Figure 3a). We consider the occurrence of direct optical transitions and determine the corresponding bandgap values in the entire compositional range investigated from the squared absorption coefficient spectra presented in Figure S5. Representative squared absorption coefficient spectra are shown in Figure 3b. All spectra were analyzed by applying the baseline method, the validity and accurateness of which were demonstrated for films composed of mixed phases [39]. The results of the determined *E*_0_(A) and *E*_0_(B) as a function of composition are summarized in Figure 3c. At compositions around the 1:2:2 stoichiometry, we determine values of *E*_0_(A) = 1.89 eV and *E*_0_(B) = 2.6 eV. The former energy, *E*_0_(A), is attributed to the bandgap of BaCu_2_Se_2_, which is the main phase in this region where only a small amount of BaSe_3_ is present. Because of the presence of the latter secondary phase, one may be tempted to attribute the energy *E*_0_(B) = 2.6 eV to the bandgap of BaSe_3_. That is, however, not the case as the bandgap of BaSe_3_ was previously reported to be between 1.59 and 1.7(2) eV [40, 41]. The origin of the *E*_0_(B) transition at 2.6 eV is clarified by analysing its behaviour by tracking changes of *E*_0_(A) as a function of the CBC atomic ratio for Ba‐rich compositions. It can be observed that for CBC < 0.74 the *E*_0_(B) follows approximately the development of *E*_0_(A), while no additional transitions, e.g., related to the BaSe_3_ bandgap of ≈1.6 eV are observed. Despite the evolution of another secondary phase, BaSe for the CBC < 0.60, the *E*_0_(B) values remain unchanged. Transitions related to BaSe are not detected since those are expected at photon energies higher than 3.6 eV [42, 43, 44], exceeding the energy range of our measurement setup. Note that also the calculated BaSe bandgap of 2.95 eV is not detected [45]. We thus conclude that the *E*_0_(B) = 2.6 eV transitions arise from deeper valence band states of BaCu_2_Se_2_, while a direct bandgap of 1.89 eV and a corresponding absorption coefficient of ≈ 8 × 10^4^ cm^−1^ is found (see Figure 3a). The determined bandgap agrees with the value of 1.8 eV previously reported for BaCu_2_Se_2_ pellets and fine powders [19, 20]. Around 1:2:2 stoichiometry, we observe constant values of *E*_g_BaCu2Se2_ for the CBC atomic ratios between ≈0.62 and 0.75. A weak decrease down to 1.83 eV is observed for high Ba concentrations corresponding to CBC = 0.40, while the lowest energy of the BaCu_2_Se_2_ bandgap is found in Cu‐rich regions with the CBC ranging between 0.75 and 0.82. For Cu‐rich areas with CBC > 0.82, we find *E*_0_(A) and *E*_0_(B) values to correspond to the direct bandgaps of the CuSe (*E*_g_CuSe_ = 1.05 eV) [46, 47] and Cu_2_Se (*E*_g_Cu2Se_ = 2.1–2.3 eV) [48, 49, 50] binary phases, which were formerly detected in this compositional range by XRD (see previous section).

#### 2.2.2 Photoluminescence and Quasi‐Fermi‐Level Splitting

Absolute intensity PL hyperspectral imaging measurements were performed at room temperature to determine the quasi‐Fermi‐level splitting (QFLS or Δ*E*_F_) [51, 52, 53, 54]. The quasi‐Fermi level splitting allows the prediction of the maximum achievable open‐circuit voltage (*V*_oc_) of a solar cell absorber material and gives an estimation of non‐radiative recombination losses. The absolute intensity PL spectra in Figure 3d were extracted from the PL intensity image shown on the inset to this figure. As can be seen, the PL is observed only for Cu‐poor areas starting from CBC = 0.63 (P0) and extending down to CBC ≈ 0.40 (P1). The PL spectrum of the slightly Cu‐poor BaCu_2_Se_2_ (≈ 3.7% Cu deficient, see Figure S6) is found centered at a peak position P0 = 1.91 eV. The P0 peak position is in good agreement with the absorption onset determined from UV–vis measurements (see Figure S7a), clearly indicating that it originates from a band‐band transition. Analysis of the PL spectrum yields an external PL quantum efficiency (EQE_PL_) [55] of 1 × 10^−5^ for a slightly Cu‐poor composition. For the same composition, Δ*E*_F_ was determined from the PL spectrum of the film by applying a fitting procedure for the high‐energy slope similar to that previously reported for different solar cell absorber films [53, 54, 55]. We found a Δ*E*_F_ = 1.30 eV, which thus implies a maximum *V*_oc_ of the same magnitude for an ideal solar cell. The energy of the P0 peak is found to decrease to 1.88 eV in highly Cu‐poor regions (see the peak analysis in Figure S7b). Both P0 values of 1.88 and 1.91 eV are in very good agreement with the development of the BaCu_2_Se_2_ bandgap found from the analysis of the UV–vis measurements in the previous section. The intensity of the P0 peak decreases by a factor of two in highly Cu‐poor/Ba‐rich areas compared to the 1:2:2 stoichiometric area, while another peak P1 evolves at an energy centered at 1.76 eV (see Figure 3d and Figure S7b). Consequently, an energy difference of 120 meV is found with respect to the BaCu_2_Se_2_ bandgap in this compositional range. An activation energy of ≈160 meV was reported in literature as deduced from temperature‐dependent conductivity measurements of *p*‐type Cu‐poor BaCu_2‐_ *_x_* Se_2_ pellets (0.01 ≤ *x* ≤ 0.04) without having been assigned to a certain type of lattice defect [23]. We note that this activation energy cannot be attributed to Cu vacancies (*V*_Cu_) in BaCu_2_Se_2,_ which previously have been reported to be shallow acceptor (A1) with activation energies ≈7 meV [19]. The formation energy of *V*_Cu_ was found to be the lowest among the formation energies calculated for several different intrinsic defects in this material [23]. The activation energy of 120 meV cannot be also attributed to another potential deeper acceptor (A2) associated with a Cu_Ba_ antisite defect, which was predicted to form in BaCu_2_Se_2_ at moderate energies [23]. That is because Cu_Ba_ antisite defects are expected to form under Cu‐rich conditions. We propose, therefore, that under Ba‐rich conditions donor states (D1) are formed, which are associated with a donor level, and therefore attribute the P1 peak to donor‐to‐valence‐band (D1‐*h*) transitions. An increase of the concentration of the D1 states with Ba‐content would lead to compensation of the *p*‐type material, and thus, to the decrease of the PL intensity, which is indeed observed in Figure 3d. A decreasing carrier density with increasing Ba‐content is also observed from electronic measurements, as will be discussed in the next section. To further clarify the nature of this defect state, temperature and intensity‐dependent PL measurements should be performed in future studies. Since its energy is very close to the reported band gap of 1.7 eV for BaSe_3_ [41], which is found as a secondary phase in this compositional range, the assignment of P1 to band‐band transitions in BaSe_3_ cannot be excluded.

### 2.3 Electronic and Transport Properties

#### 2.3.1 Conductivity Type and Charge Carrier Concentration by KP‐PYS

Surface electronic properties of the Ba‐Cu‐Se combinatorial films were investigated by combining Kelvin probe (KP) measurements and photoelectron yield spectroscopy (PYS) under inert N_2_ atmosphere at ambient pressure [56]. By these methods, we have determined important material electronic parameters such as the work function (WF or Φ), which defines the Fermi level (*E*_F_), and the ionization energy (IE or *E*_i_), which is directly related to the energy of the valence band maximum (*E*_VBM_). With the known bandgaps, one determines the conduction band minimum (CBM) energy *E*_CBM_ = *E*_VBM_ + *E*_g_ and thus the electron affinity (EA). These electronic parameters are of importance for obtaining the band diagram of the investigated materials, which are then used in simulations of solar cell PV parameters.

**FIGURE 4:**
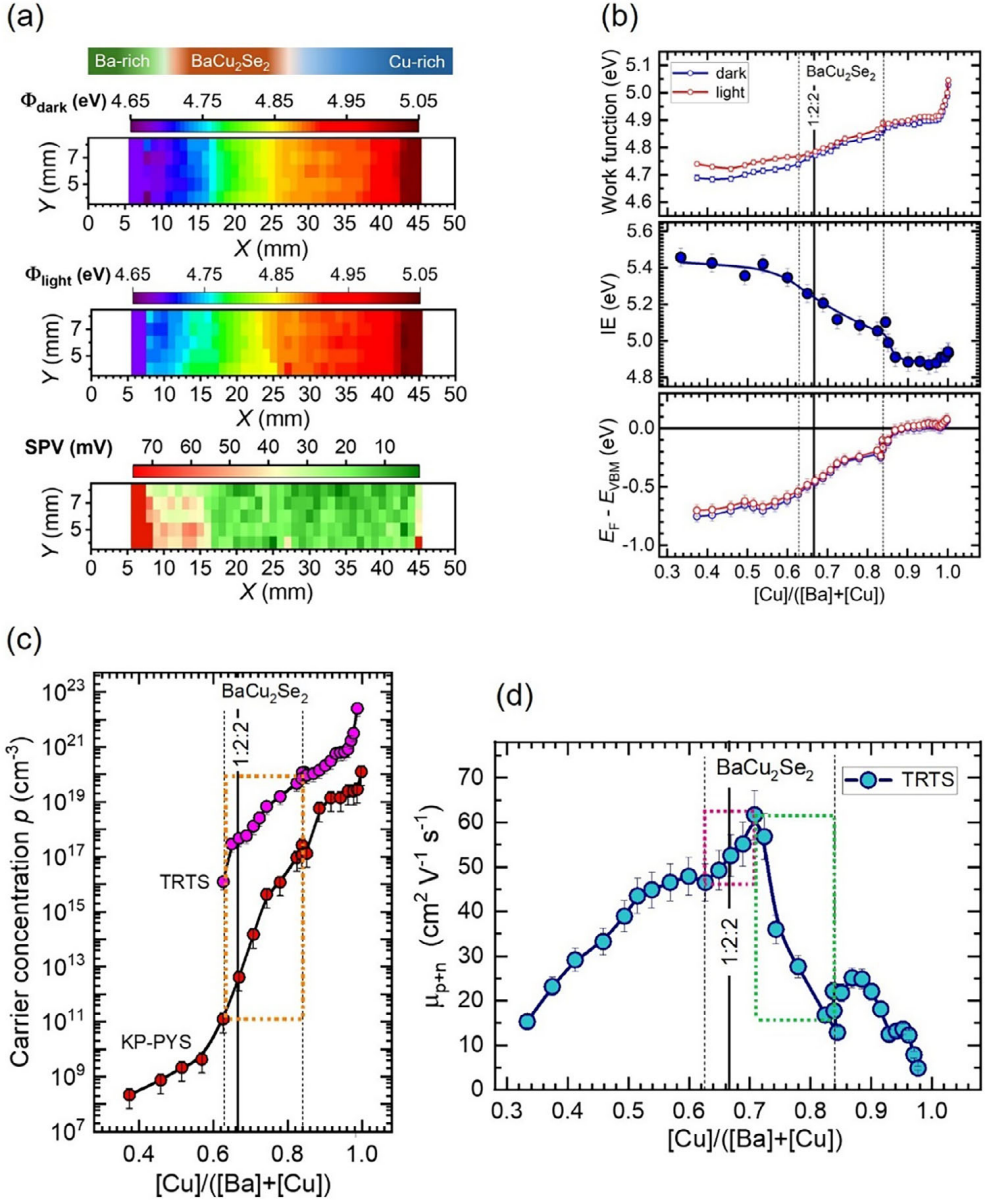
Electronic properties of Ba‐Cu‐Se combinatorial films. (a) Surface work function (Φ) maps obtained from Kelvin probe (KP) measurements at room temperature under N_2_ gas at ambient pressure. The measurements under illumination were performed with a cw laser at 450 nm / 2.75 eV at a power density of 100 mW/cm^2^. The surface photovoltage (SPV) maps are calculated as Φ_light_−Φ_dark_. (b) Line‐scans along the middle line of the sample (*Y* = 6 mm) of the work function (WF) (top panel), ionization energy (IE) (middle panel) and the calculated valence band maximum (*E*_VBM_) with respect to the Fermi level (*E*_F_) as function of [Cu]/([Ba]+[Cu]) ratio. (c) Concentration of holes, *p*, as calculated from combined KP and photoelectron yield spectroscopy (KP‐PYS), and time‐resolved terahertz spectroscopy (TRTS) data as a function of [Cu]/([Ba]+[Cu]) ratio. The dotted orange rectangle depicts the dependence of the charge carrier concentration on film composition in α‐BaCu_2_Se_2_ phase from Cu‐poor/Ba‐rich to Cu‐rich/Ba‐poor. (d) The hole and electron sum mobility (*µ*_p+n_) as a function of thin film composition determined from TRTS measurements. The pink and green dotted rectangles show the regions of the mobility increase and decrease, respectively, which cover the compositional range where the α‐BaCu_2_Se_2_ phase dominates. The continuous vertical lines at [Cu]/([Ba]+[Cu]) = 0.67 show the reference 1:2:2 stoichiometry.

Work function mappings of the combinatorial Ba‐Cu‐Se library in the dark and under illumination (see Figure 4a) reveal Φ values varying between 4.7 and 5.0 eV, which correspond to material phases distributed along the sample. For the near 1:2:2 stoichiometry, a work function in the dark of 4.75 eV was measured. By subtracting the Φ values of the mappings in the dark from those under illuminated conditions (Φ_light_−Φ_dark_), we obtain the mapping of the surface photovoltage (SPV). All the obtained SPV values are positive and thus demonstrate a *p*‐type conductivity of all the observed phases. Low SPV values detected for the BaCu_2_Se_2_ phase are explained by a high concentration of surface defects, which lead to Fermi level pinning. The SPV amplitude increases with the Ba concentration, indicating a lower concentration of surface defects, which correlates well with the observed behaviour of the PL signals from regions with Ba‐rich composition. A very low SPV is detected from Cu‐Se phases, as those are in a degenerate state, as will be shown below. IE was scanned only along the middle line of the sample, since a uniform lateral distribution of the surface work function was recorded. The IE dependence on the CBC atomic ratio in Figure 4b (middle panel) is derived from PYS spectra shown in Figure S8. It shows large variations along the sample, changing from about 5.4 eV for CBC ≈ 0.40 on the Ba‐rich side to about 4.9 eV for CBC ≈ 0.98 on the Cu‐rich side. The ionization energy of the 1:2:2 stoichiometric BaCu_2_Se_2_ is found to be 5.22 eV. Considering the bandgap of 1.89 eV as determined from UV–vis data, we determine the electron affinity EA = 3.33 eV. For comparison, DFT calculations give *E*_i_ = 4.50 eV and EA = 3.17 eV [24]. With the WF and IE data measured along the same line scan, we calculate the VBM position with respect to the Fermi level as shown in Figure 4b (bottom panel). Cu‐rich films with a CBC > 0.90, which contain only the CuSe and Cu_2_Se phases (see Figure 2b), are found to be in the degenerate state as the Fermi level lies on the VBM or even goes slightly into valence band. An almost gradual increase of the *E*_F_−*E*_VBM_ is found with the Ba content increase. For the 1:2:2 stoichiometric BaCu_2_Se_2_ we calculate an *E*_F_−*E*_VBM_ = 0.45 eV. We further use the *E*_F_−*E*_VBM_ data for calculation of the equilibrium carrier concentration, *p*, as a function of the CBC atomic ratio. We apply Boltzmann statistics for *p*‐type semiconductors according to which the concentration of holes $p=N_{V}\;\exp\left[-(E_{F}-E_{VBM})/kT\right]$, where *N_V_* ≡ 2(2π*m_h_kT*/*h* ^2^)^3/2^ is the effective density of states in the valence band, *k* is the Boltzmann constant, *T* is the absolute temperature, *m_h_* is the density‐of‐state effective mass of holes in the valence band and *h* is the Planck constant [57]. We consider for the regions where the α‐BaCu_2_Se_2_, Cu_2_Se, and CuSe phases dominate the effective masses of holes *m_h_*__α‐BaCu2Se2_ = 3.14*m*_o_ [25], *m_h_*__Cu2Se_ = 0.40*m*_o_ [58] and *m_h_*__CuSe_ = 0.25*m*_o_ [59], respectively, and plot the resulting *p* data as a function of atomic composition in Figure 4c. The equilibrium near‐surface concentration of holes for the 1:2:2 stoichiometry of the *α*‐BaCu_2_Se_2_ phase is found to be 4.2 × 10^12^ cm^−3^, while linearly varying between 2.7 × 10^10^ cm^−3^ and 1.2 × 10^16^ cm^−3^ for the CBC atomic ratios changing between 0.60 and 0.78, i.e., from Cu‐poor to Cu‐rich compositions. We thus find that the charge carrier concentration in the *α*‐phase BaCu_2_Se_2_ increases with increasing Cu concentration. This result appears to be in contradiction with a recent study, which demonstrated that Cu vacancies are responsible for the high concentration of holes in this material [23]. In this publication, though, the Cu content in BaCu_2‐x_Se_2_ was only varied at a fixed Ba concentration. In our study, however, the Ba concentration varies from an excess of ≈5 at.% in Cu‐poor BaCu_2_Se_2_ with a CBC = 0.60 to a deficiency of ≈9 at.% in Cu‐rich BaCu_2_Se_2_ with a CBC = 0.78. As discussed in Section 2.2.2, excess Ba in Cu‐poor BaCu_2_Se_2_ can provide the formation of deep D1 donors. Thus, those donors will compensate the shallow acceptors A1 related to *V*_Cu_, which were introduced above for the explanation of the PL spectra. Therefore, the low concentration of holes in Cu‐poor BaCu_2_Se_2_ is explained by the compensating effect of the donors on acceptors. This finding agrees well with the lowered PL intensity in Cu‐poor regions (see Figure 3d). In Ba‐poor and Cu‐rich BaCu_2_Se_2_, we expect the formation of additional acceptors, namely, A2 related to *V*_Ba_ and A3 associated to Cu_Ba_ antisite defects stimulated by a large Ba deficiency [23, 27]. Thus, the appearance of the A2 and A3 acceptors shall be supplemented by a significant increase of the concentration of holes. This increase should be even more supported when considering that with the increase of the Cu content, accompanied by a simultaneous decrease of the Ba content, the concentration of the compensating D1 donors shall decrease. Thus, the significant increase in the concentration of holes in the Cu‐rich BaCu_2_Se_2_ is explained by the development of the A2 and A3 acceptors along with a continuous reduction of the compensation due to a constantly decreasing concentration of the D1 donor. It can be concluded, therefore, that a significant increase of the concentration of holes in BaCu_2_Se_2_ by a few orders of magnitude can be achieved by engineering *V*_Cu_ in conjunction with *V*_Ba_ and Cu_Ba_ antisite defects via lowering the Ba content.

#### 2.3.2 Charge Carrier Concentration, Lifetime, and Mobility by TRTS and OPTP

Charge carrier concentrations *p*, hole and electron sum mobilities *µ_p+n_*, and charge carrier decay time *τ*, were investigated by time‐resolved THz spectroscopy (TRTS) and time‐resolved optical‐pump terahertz‐probe (OPTP) spectroscopy, which allow to characterize the charge carrier dynamics with high temporal resolution [60, 61, 62, 63]. Note that in polycrystalline thin films, the measured THz mobilities and concentration of charge carriers are intragrain values [61, 62, 63]. The intragrain charge carrier concentration inferred from THz absorption is presented in Figure 4c, which shows a similar trend with the Cu concentration as it was found from KP‐PYS analysis of the surface electronic properties, although at significantly larger values. Thus, significant differences between near‐surface and intragrain doping concentrations are observed. In general, from KP‐PYS data, one can determine doping concentrations of the film bulk, which should be comparable to intragrain doping determined from TRTS. However, the estimation of the bulk carrier concentration in BaCu_2_Se_2_ films from KP‐PYS measurements was impeded by the fact of the Fermi level pinning at the film surface (see Section 2.3.1). Due to Fermi level pinning in the near‐surface region of the film, the work function of the bulk cannot be obtained by correcting the measured WF under illumination for surface potential barrier height, since no flat band conditions could be achieved. At surfaces with non‐pinned Fermi levels, potential barrier heights between 100 and 200 meV could usually be determined. In terms of charge carrier concentrations, this will result in differences between the bulk and surface doping levels of about two to four orders of magnitude. That explains the differences observed between charge carrier concentrations determined for the near‐surface and intragrain regions in the studied BaCu_2_Se_2_ thin films. By means of THz spectroscopy, we find for the stoichiometric BaCu_2_Se_2_ phase a carrier concentration *p* = 2.9 × 10^17^ cm^−3^, which is in close agreement with the previously published data on pellets [19, 20, 23]. The concentration of holes varies as a function of CBC atomic ratio between 2.9 × 10^16^ and 1.6 × 10^19^ cm^−3^ for the CBC variation between 0.63 and 0.78. The latter carrier concentration is very close to an optimum of 6.3 × 10^19^ cm^−3^ calculated for achieving the maximum power factor (*PF*) needed for application in TE [23]. The hole and electron sum mobilities *µ*_p+n_, obtained from OPTP measurements, are shown in Figure 4d as a function of composition. For strongly Cu‐poor *α*‐BaCu_2_Se_2_, values of ≈ 20 cm^2^ V^−1^ s^−1^ are observed, which increase monotonically with Cu‐content until maximum values of 55–60 cm^2^ V^−1^ s^−1^ are reached close to the stoichiometric 1:2:2 region. A further increase of the CBC ratio beyond 0.71 leads to an abrupt decrease of mobility down to 20 cm^2^ V^−1^ s^−1^, further decreasing for very high CBC close to 1. Comparing our results with literature values, we note that the *µ*_p+n_ of *α*‐BaCu_2_Se_2_ thin films are by a factor of three higher compared to those previously reported on pelletized powders [19, 20, 23]. This can be due to several reasons. First, mobility values from THz measurements, as it was pointed out above, estimate the intragrain mobility which is not limited by grain boundary scattering [60, 62, 64], and second, the THz‐absorption derived mobilities give the sum mobility of electrons and holes, while the hole measurements reported in the literature are sensitive only to majority carriers, holes in the case of BaCu_2_Se_2_. The effective bulk lifetime of carriers was determined from decays in the THz photoconductivity transients measured on different sample positions with different CBC atomic ratios (see Figure S9a). For the stoichiometric composition, a decay time *τ* ≈ 7 ps is observed, while larger and smaller CBC ratios lead to shorter decay times. The decay time could correspond to a trapping time into defects or represent a rather low recombination lifetime of photogenerated carriers in the material. Since the photoluminescence quantum yield is directly related to the doping density and recombination lifetime, the three measurement results can be checked for consistency. Such analysis shows (see Figure S9b) that indeed the currently observed recombination lifetime in the BaCu_2_Se_2_ thin films is likely on the picosecond time scale, indicating significant non‐radiative recombination by defects which would have to be reduced for future application in solar cells.

#### 2.3.3 Electrical Conductivity and Thermoelectric Properties

**FIGURE 5:**
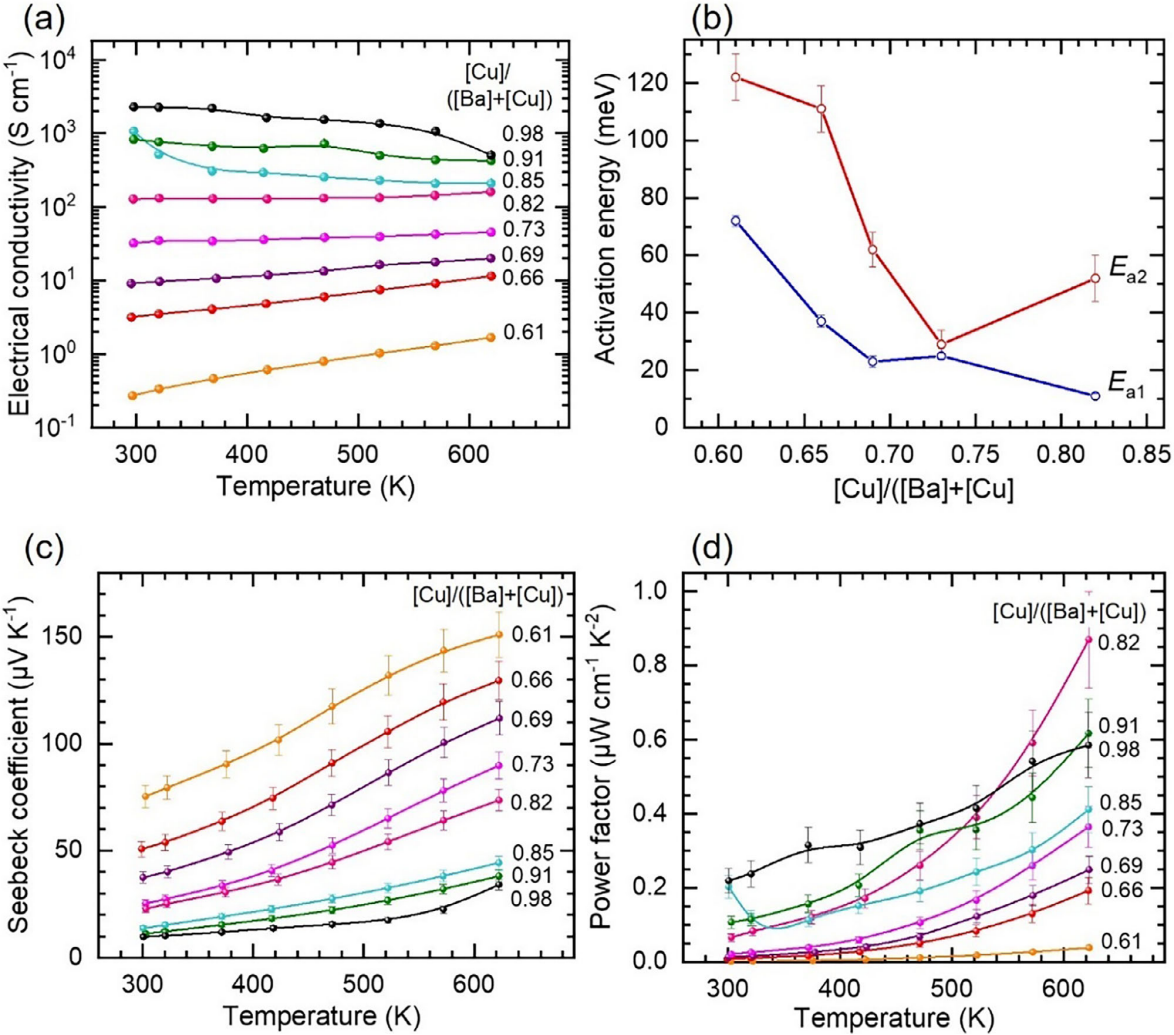
Electrical conductivity and thermoelectric performance of Ba‐Cu‐Se combinatorial films. (a) Temperature dependence of electrical conductivity as a function of [Cu]/([Ba]+[Cu]) atomic ratio. (b) Activation energies *E*_a1_ and *E*_a2_ of the electrical conductivity versus chemical composition as obtained from Arrhenius plots in Figure S8 from fits in the low and high temperature ranges, respectively. Temperature dependencies of (c) Seebeck coefficient and (d) power factor for various [Cu]/([Ba]+[Cu]) atomic ratios.

To estimate the ability of *α*‐BaCu_2_Se_2_ to generate electrical power from temperature differences, we have studied its power factor, which combines the electrical conductivity, *σ*, and Seebeck coefficient, *S*, as *PF* = *σ*S^2^ [28, 65]. The conductivity and Seebeck coefficient were investigated as a function of CBC atomic ratio in the temperature range between 295 and 623 K by a commercial Seebeck analyzer (SBA 458 Nemesis from NETZSCH) under Ar atmosphere (5N purity) at ambient pressure. The electrical conductivity presented in Figure 5a shows for the *α*‐phase BaCu_2_Se_2_ with the 0.61 ≤ CBC ≤ 0.82 an exponential increase with temperature, which is typical for semiconductor behavior with a thermally activated increase in charge carrier concentration. In contrast, the electrical conductivity decreases with temperature for CBC ≥ 0.85, indicating metallic behavior with negligible variations in charge carrier concentration but increased electron‐phonon scattering at elevated temperatures. These observations agree with the results from KP‐PYS, which showed that the CuSe and Cu_2_Se binaries observed in this compositional range are degenerate (see §2.3.1). The electrical conductivity of *α*‐BaCu_2_Se_2_ shows an exponential Arrhenius‐type temperature dependence σ  = σ_0_ *exp*(− Δ*E_a_*/*kT*), where *σ*_0_ is the nominal conductivity at infinite temperature, *E*_a_ is the activation energy for conductivity, and *k* is the Boltzmann constant (see Figure S10) [66]. Linear *ln σ* vs 1/*T* dependencies are observed in the low and high temperature regions, and thus two activation energies, *E*_a1_ and *E*_a2_, can be determined. The dependencies of *E*_a1_ and *E*_a2_ on the CBC atomic ratio are presented in Figure 5b. *E*_a1_ is found to decrease from 71 ± 2 meV for CBC = 0.61 to 11 ± 1 meV for CBC = 0.82. *E*_a2_ varies from 122 ± 8 meV at CBC = 0.61 to 52 ± 8 meV at CBC = 0.82. For the 1:2:2 stoichiometry, *E*_a1_ = 40 ± 2 meV and *E*_a2_ = 112 ± 8 meV.

Supported by the discussion of our PL and KP‐PYS results, we assume *E*_a2_ to originate from Cu_Ba_ antisite defects. The development of *E*_a1_ likely points to the activation of the energy level A1 attributed to *V*_Cu_ defects, which have the lowest calculated formation energy in α‐BaCu_2_Se_2_ [23].

Dependences of the Seebeck coefficient on temperature for various CBC ratios in Figure 5c show positive values in the entire investigated compositional range, which indicate *p*‐type conductivity of the films. This result confirms the conclusion on the *p*‐type conductivity based on the SPV and calculated *E*_F_‐*E*_VBM_ energy from KP‐PYS measurements. The highest Seebeck coefficient of ≈150 µV K^−1^ is achieved for films with CBC = 0.61. The calculated *PF* for this composition, however, shows minimum values (see Figure 5d) due to the low electrical conductivity (see Figure 5a). The highest *PF* is achieved at 623 K for BaCu_2_Se_2_ films with the CBC = 0.82, characterized by a high conductivity with the lowest activation energy *E*_a1_ = 11 ± 1 meV and *E*_a2_ = 52 ± 8 meV. The films with the CBC > 0.82, which are composed of degenerate CuSe and Cu_2_Se, show higher *PF*s at room temperature than BaCu_2_Se_2_. However, at temperatures > 580 K, the *PF* of BaCu_2_Se_2_ surpasses the *PF* of Cu‐Se binaries, reaching 0.87 µW cm^−1^ K^−2^ and thus becomes comparable with the data published on hot‐pressed BaCu_2_Se_2_ samples [23].

## 3 Conclusions

In conclusion, we have prepared BaCu_2_Se_2_ thin films, which were synthesized at 540 °C in vacuum by selenization of Cu‐BaO precursors deposited by PLD on quartz glass substrates (12 mm × 50 mm). By realizing a compositional gradient along substrates, combinatorial thin film libraries were created with [Cu]/([Ba]+[Cu]) atomic ratios spreading over a range of 0.15–0.98.

Structural analysis revealed formation of a BaCu_2_Se_2_ thin film of the stable orthorhombic *α*‐phase (space group *Pnma*) with the unit cell constants of *a* = 9.594 (2) Å, *b* = 4.214 (1) Å, and *c* = 10.771 (2) Å. The *α*‐BaCu_2_Se_2_ phase is found to form and dominate for [Cu]/([Ba]+[Cu]) atomic ratios between 0.15–0.85 and 0.62–0.82, respectively. On the Ba‐rich sample side, along with the *α*‐BaCu_2_Se_2_ phase, BaSe and BaSe_3_ secondary phases are present. BaCu_2_Se_2_ films with the stoichiometry close to 1:2:2 contain a small fraction of BaSe_3_ phase (≤ 7 wt.%). CuSe and Cu_2_Se phases evolve for [Cu]/([Ba]+[Cu]) ≥ 0.80.

Optical measurements revealed for the stoichiometric BaCu_2_Se_2_ a direct optical band gap with an energy of 1.89 eV and an absorption coefficient of 8 × 10^4^ cm^−1^. The bandgap is found remarkably stable within the [Cu]/([Ba]+[Cu]) compositional range of 0.62–0.75. Slightly lower bandgap values are observed for Ba‐rich compositions. The lowest energy of the BaCu_2_Se_2_ bandgap is found for Cu‐rich compositions with [Cu]/([Ba]+[Cu]) ≥ 0.80. Absolute PL hyperspectral imaging revealed a bright luminescence centered at 1.91 eV, which is close to the bandgap energy and is thus attributed to band‐band transitions. The VBM position is found from PYS at 5.20 eV below the vacuum level. By considering the bandgap energy, the EA is calculated to be 3.33 eV. For the quasi‐Fermi‐level splitting under one sun illumination, a maximum value of 1.30 eV is determined, which implies for solar cells from *α*‐BaCu_2_Se_2_ absorbers a maximum theoretically achievable *V*_oc_ of a similar magnitude.

As found from the SPV and Seebeck coefficient measurements, *α*‐BaCu_2_Se_2_ thin films are of *p*‐type conductivity irrespective of the [Cu]/([Ba]+[Cu]) atomic ratio. The KP‐PYS and TRTS measurements have demonstrated that the equilibrium concentration of holes can be tuned over a few orders of magnitude just by varying the atomic concentrations of Cu and Ba. The intragrain concentration of holes can be varied from ∼10^16^ cm^−3^ at [Cu]/([Ba]+[Cu]) ≈ 0.62 to ∼10^20^cm^−3^ at [Cu]/([Ba]+[Cu]) ≈ 0.84. These strong variations in carrier concentrations are attributed essentially to the competitive activation of a few acceptor levels and one donor level, which form predominantly under Ba‐rich and Cu‐poor conditions. From PL measurements, the activation energy of the donor is calculated to be ≈120 meV. A shallow acceptor should be attributed to *V*_Cu_, which has the lowest calculated formation energy among different intrinsic defects in BaCu_2_Se_2_. Additional potential acceptor levels should originate under Ba‐poor and Cu‐rich conditions from *V*_Ba_ and Cu_Ba_ antisite defects. Charge carrier mobilities show a strong dependence on chemical composition, reaching a maximum value of ≈60 cm^2^ V^−1^s^−1^ at [Cu]/([Ba]+[Cu]) ≈ 0.70.

The highest *PF* for generating electrical power from temperature differences is found to be 0.87 µW cm^−1^ K^−2^ at a temperature of 623 K for [Cu]/([Ba]+[Cu]) ≈ 0.82.

Thus, tunability in a large range of the electronic properties of BaCu_2_Se_2_ by a controlled adjustment of the [Cu]/([Ba]+[Cu]) atomic ratio makes this material very attractive for application (i) in photovoltaics when prepared under Ba‐rich/Cu‐poor conditions and (ii) in thermoelectrics when prepared under Cu‐rich/Ba‐poor conditions.

## 4 Experimental Section

### 4.1 Thin Film Preparation

BaCu_2_Se_2_ combinatorial thin films were synthesized by applying a two‐stage preparation technology. In the first stage, BaO and Cu wedge layers were prepared. In the second stage, the BaO and Cu precursors were selenized. The wedge precursors of BaO and Cu layers were prepared by a pulsed laser deposition (PLD) system from Surface systems+technology GmbH. BaO and Cu wedge films were deposited on 12 × 50 mm^2^ sized quartz glass substrates at room temperature by scanning the laser beam from a KrF excimer laser (Coherent Laser Systems, COMPex 205F, 248 nm wavelength, 30 ns pulse duration) on 2‐inch diameter rotating targets of respective materials acquired from Plasmaterials, Inc. The wedge‐shaped films were obtained by software‐controlled movement of substrates behind a slit with a width of 10 mm. The ablation of materials was performed at a background pressure of 5.0 × 10^−4^ mbar. The deposition pressure was maintained constant by a controlled inlet of N_2_ gas (5N purity). The laser fluences were set at 1.0 and 7.0 J/cm^2^ for BaO and Cu, respectively. Under these preparation conditions, the deposition rate of BaO was 1.92 Å/pulse and the deposition rate of Cu was 0.81 Å/pulse.

The BaO and Cu precursor layers were selenized in a physical vapor deposition (PVD) system at the substrate temperature T_substrate_ = 540°C for 5 min at a pressure of 2 × 10^−6^ mbar from a selenium source heated to T_Se_ = 350°C. The substrate temperature was chosen according to the revised in the Introduction literature data for obtaining the stable α‐phase BaCu_2_Se_2_. The selenization time was set based on our previous studies on elemental depth distribution and formation of crystalline phases during selenization of layered precursor stacks for the formation of chalcogenide material systems. (67) The base pressure in the PVD system was 1 × 10^−7^ mbar. The ramp temperature was 1.6 K/sec. The cooling rate to approximately room temperature was ≈0.14 K/sec.

### 4.2 XRF Analysis

XRF analysis was applied for the determination of the elemental composition and thicknesses of the films. The XRF was performed by using a Bruker M4 Tornado with a Rhodium X‐ray tube operated at 200 µA and 50 kV and one detector. The samples were measured under a vacuum of ≈1mbar. A polycapillary optic was used to focus the X‐ray beam down to a spot size of 20 µm. The mapping measurements were performed along three‐line scans of 25 points each, with 1 mm spacing along the compositional gradient and with 2 mm spacing between the line scans.

### 4.3 GIXRD Analysis

GIXRD measurements were conducted with an X‐Ray spot size of about 3 mm along a linear scan on 17 points at an incident angle of 3° with a step of 3 mm to study the crystal structure and the phase content of the films. The data were collected using a PANalytical X'pertPro MPD diffractometer equipped with a CuKα radiation source (λ = 1.54056 Å). Detector scans were obtained with a collection time of 7 s and a step size of 0.04°. Additionally, GIXRD measurements with different incident angles (0.3°, 0.5°, 1 °, and 2°) were conducted on a sample position corresponding to the 1:2:2 of the BaCu_2_Se_2_ stoichiometry with a collection time of 30 s, and a step size of 0.04°.

### 4.4 Scanning Electron Microscopy (SEM) and Energy Dispersive X‐Ray Spectroscopy (EDS)

SEM images were acquired using a Zeiss Leo 1530 Gemini scanning electron microscope operated at acceleration voltages between 3 and 0 kV. EDS was applied for the determination of local elemental compositions by using a Thermo Scientific UltraDry system within the SEM microscope. The detection of the elemental lines, analysis of the data, and recording of the elemental mappings were performed by employing the software COMPASS by Thermo Fisher Scientific.

### 4.5 UV–vis Spectroscopy

Optical transmittance (T) and reflectance (R) of the thin films were measured at room temperature with a light beam of 2 × 10 mm^2^ spot size in a Perkin Ellmer Lambda 750 spectrometer in combination with an integrating sphere in order to collect also the scattered light. The transmittance was measured under vertical incidence, and the reflectance under slightly tilted incidence to collect the directly reflected light. Analysis of the transmittance and reflectance data was used for the evaluation of the absorption coefficient, as well as for the identification of different energy transitions.

### 4.6 Absolute Intensity Photoluminescence Hyperspectral Imaging

Absolute intensity PL measurements were performed by using a custom setup described elsewhere [53, 54, 55, 68]. In the present study, the samples were completely illuminated with two 450 nm LEDs equipped with diffuser lenses. The intensity of the LEDs was 1.6 × 10^21^ photons m^−2^ s^−1^. The photoluminescence was recorded as a complete image with a charge‐coupled device (CCD) camera. The spectral resolution at each pixel was obtained by using a spectral filtering setup with a liquid crystal tunable filter. The pixel resolution of PL images corresponds to ≈10 µm in diameter. PL images were recorded in a wavelength range of 500–1100 nm at a step size of 5 nm. The system was calibrated to absolute photon numbers and thus allowed to determine the quasi‐Fermi‐level splitting (QFLS) [51, 52]. The QFLS was determined from the evaluation of the high‐energy slope of spatially integrated PL spectra [52, 53, 54] on areas of 2 × 10 mm^2^.

### 4.7 Combined Kelvin Probe and Photoelectron Yield Spectroscopy

Kelvin probe (KP) and photoelectron yield spectroscopy (PYS) were combined to measure the work function (Φ) and ionization energy (E_i_) of the prepared thin films, respectively, by employing a KP Technology SKP5050‐APS02 setup under an ambient pressure environment of nitrogen gas at room temperature [56]. The setup is enclosed in a Faraday cage to shield external electric fields and enable controlled illumination of the sample. The samples were contacted with carbon tape. The Kelvin tip was a 2.0 mm diameter electrode with a gold‐alloy coating with a work function, Φ_tip_, calibrated against a gold reference sample. The work function of the sample, Φ_sample_, was determined according to Φ_sample_ = Φ_tip_ + e·CPD where e is the elementary charge and CPD is the contact potential difference between the Kelvin tip and the investigated sample. The CPD was measured with a resolution of 1–3 mV. The measurements of the surface photovoltage (SPV) defined as SPV = CPD_light_ − CPD_dark_ were performed using a cw laser diode with a wavelength/energy of 450 nm/2.75 eV with a light power density of ≈100 mW/cm^2^. PYS measurements were performed by using the same Kelvin tip in the static regime. The sample was illuminated by a deuterium light source coupled with a grating monochromator, providing excitation from 3.4 to 7.6 eV. Measurements were conducted with a step of 1 nm, and the photoemission threshold was determined with a resolution of 30 meV.

### 4.8 Time‐Resolved THz Spectroscopy (TRTS) and Optical Pump THz‐Probe (OPTP) Spectroscopy

TRTS and OPTP spectroscopies based on an amplified Ti‐Sapphire laser system were employed to determine the mobility, concentration, and lifetime of charge carriers [61, 62]. The sample was placed within a N_2_ flooded box in which the THz setup was aligned. The measurements were performed at room temperature. Charge carriers were photo‐excited by a 400 nm pump pulse with 50 fs pulse width and a repetition rate of 150 kHz, and a power density of 46 mW/cm^2^. The induced change in the transmission of a terahertz probe pulse was measured by electro‐optical sampling in a 1 mm thick ZnTe crystal. This change was modelled by the thin film approximation method, which yields the photoconductivity and subsequently the sum mobility of excited electrons and holes. The transient scans by OPTP were performed at the maximum of the THz pulse. The THz probe spot size on the film surface was ≈1mm and thus averaged the mobility over the respective area. The error in the extracted mobility is estimated to be ≈20% and consists mainly of uncertainties in the excited carrier concentrations, the layer thicknesses, and the refractive indices as input for the transfer matrix analysis, as well as errors from the DC‐mobility fit. The concentration of charge carriers was determined from calculations within injection‐dependent modelling of TRTS‐transients [62]. Further details of the setup can be found elsewhere [69].

### 4.9 Thermoelectric Analysis

The simultaneous determination of the electrical conductivity and Seebeck coefficient was conducted under Ar atmosphere (5N purity) at ambient pressure by an SBA 458 Nemesis Seebeck analyzer purchased from NETZSCH. Electrical conductivity was determined by the 4‐point probe method. The outer contacts were used to provide the current, and the two center thermocouples (NiCr and NiAl) were used to record the sample temperature and measure the voltages between them, U_A_ and U_B_, which are determined between the two positive and two negative thermocouple wires. U_A_ and U_B_ were measured several times at each sample temperature for various current values and both polarities. With the known film thickness and distance between contacts, the electrical conductivity was determined from the obtained linear I‐U dependencies.

For the determination of the Seebeck coefficient, temperature gradients were generated by two micro heaters placed below the sample edges. The micro heaters were operated in alternation to create temperature gradients in both sample directions. The resulting voltages U_A_ and U_B_ were measured during heating cycles and plotted as a function of temperature difference ΔT determined between two thermocouples for both sample directions. The Seebeck coefficient was then determined from the slope of the obtained U‐ΔT linear dependencies.

The measurements were conducted at each sample position at maximum currents of 10 mA and taking into account a rectangular geometry of 12.7 mm × 1.0 mm. Correction factors were obtained from fitting the electrical conductivity at room temperature to the data gained from TRTS measurements. The accuracy of the measurements was ± 5% for electrical conductivity and ± 7% for the Seebeck coefficient.

## Conflicts of Interest

The authors declare no conflicts of interest.

## Supporting information




**Supporting File**: smll73267‐sup‐0001‐SuppMat.pdf.

## Data Availability

The data that support the findings of this study are available from the corresponding author upon reasonable request.
