# Supplementary material for: Combinatorial Synthesis of BaCu2Se2 Thin Films: Effect of Composition on Crystal Structure and Optoelectronic Properties
Source: Small. 2026 Apr 2;22(29):e73267. doi: 10.1002/smll.73267 (PMC13206570; doi:10.1002/smll.73267)
Supplement: Supplementary file 1 — Supporting File: smll73267‐sup‐0001‐SuppMat.pdf. [file SMLL-22-e73267-s001.pdf]

Supporting Information

Combinatorial Synthesis of $BaCu_2Se_2$ Thin Films: Effect of Composition on Crystal Structure and Optoelectronic Properties

Marin Rusu,[1] José A. Márquez,[1] Hannes Hempel,[1] Galina Gurieva,[1] Leo Choubrac,[1] Ibrahim Simsek, Rene Schwiddessen,[1] Pablo Reyes-Figueroa,[1] Daniel Molpeceres,[1] Robert Wenisch,[1] Markus Schleuning,[1] Jan-Ekkehard Hoffmann,[1] Klaus Habicht,[1,2] Christian A. Kaufmann,[1] Iver Lauermann,[1] Susan Schorr[1,3] and Thomas Unold[1]*

[1]Helmholtz-Zentrum Berlin für Materialien und Energie GmbH, Hahn-Meitner-Platz 1, 14109 Berlin, Germany

[2]Universität Potsdam, Karl-Liebknecht-Straße 24/25, 14476 Potsdam, Germany

[3]Freie Universität Berlin, Malteserstr. 74-100, 12249 Berlin, Germany

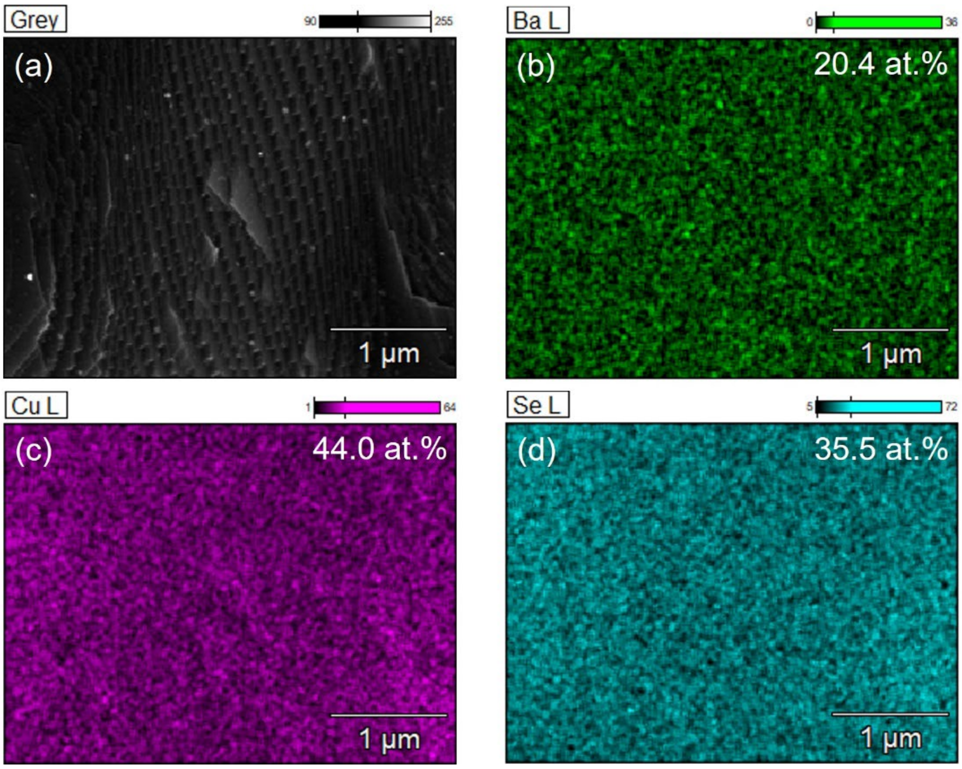

Figure S1. (a) Scanning electron microscopy (SEM) image (top view) of a 2.3 µm thick $BaCu_2Se_2$ thin film with [Cu]/([Ba]+[Cu]) = 0.68 deposited on a Mo coated soda lime glass substrate. (b–d) Energy dispersive X-Ray spectroscopy (EDS) maps corresponding to the rectangular area of the SEM image in Figure S1a.

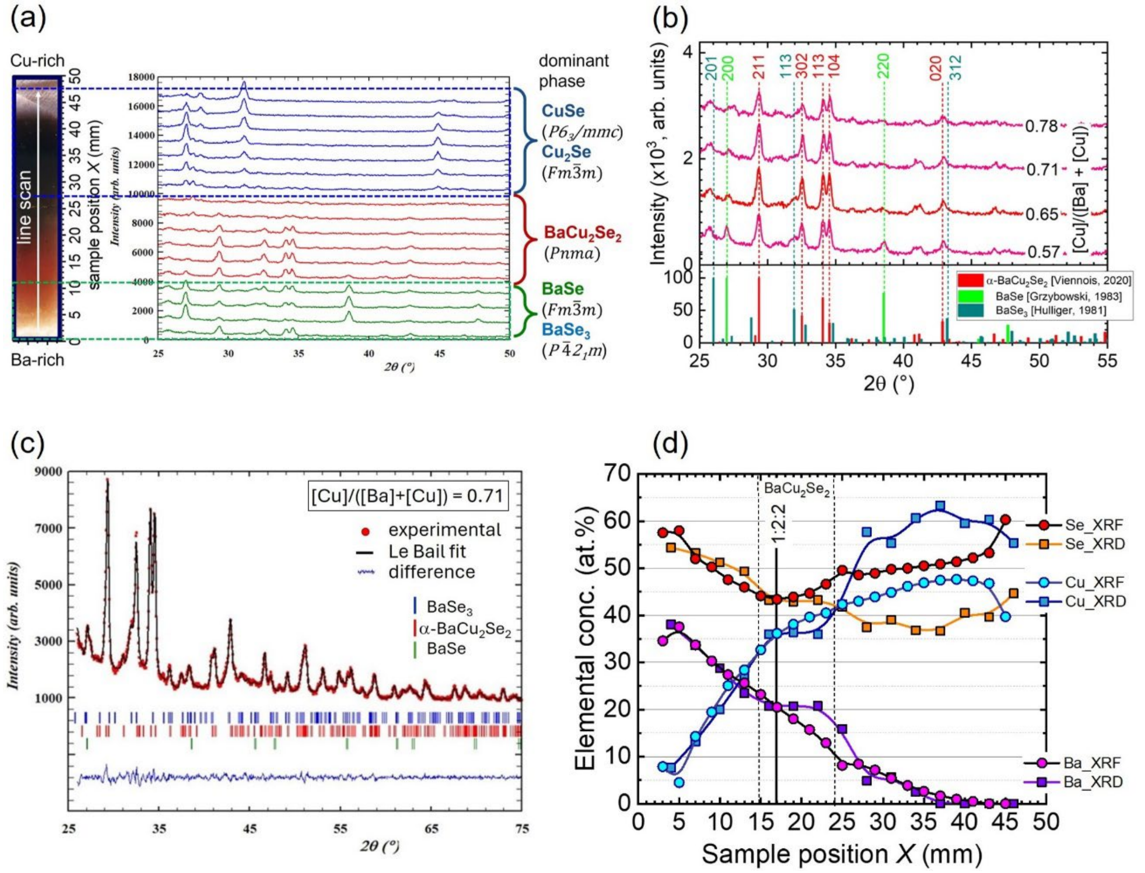

Figure S2. (a) Grazing incidence X-ray diffraction (GIXRD) patterns from the linear line-scan along the sample shown on the optical image of the Ba-Cu-Se combinatorial specimen measured at an incident angle of 3° on 17 points with a step size of 3 mm. (b) Selected GIXRD patterns from the combinatorial sample area with a composition around the 1:2:2 stoichiometry of $BaCu_2Se_2$ corresponding to variations of the [Cu]/([Ba]+[Cu]) ratio between 0.57–0.78. Note that the 1:2:2 stoichiometry is matched by [Cu]/([Ba]+[Cu]) = 0.67. (c) Le Bail refinement of the GIXRD pattern of the combinatorial film at the position #7 corresponding to $BaCu_2Se_2$ with [Cu]/([Ba]+[Cu]) = 0.71**.** The incident beam was set at an angle of 1°. The solid black line is the Le Bail fit of the experimental data (red dots) including an orthorhombic α-$BaCu_2Se_2$ phase,[1] a cubic BaSe[2] phase and a tetragonal $BaSe_3$[3] phase in the refinement. The red, green and blue bars represent the Bragg peak positions of α-$BaCu_2Se_2$, BaSe and $BaSe_3$, respectively. (d) Elemental concentration profiles calculated by considering the phase fractions found by XRD measurements and displayed in Figure 2b. The data obtained are shown in comparison with the elemental concentrations determined by X-ray fluorescence (XRF) analysis.

Table S1. Lattice parameters of the α-$BaCu_2Se_2$ phase obtained from the Le Bail refinement of GIXRD patterns of the combinatorial film with [Cu]/([Ba]+[Cu]) = 0.71. GIXRD measurements were conducted at incident angles of 0.3º, 0.5º, 1º and 2º with a collection time of 30 s and a step size of 0.04°. The values from literature are given for comparison.

**Incident angle**	***a*** **(Å)**	***b*** **(Å)**	***c*** **(Å)**
**0.3°**	9.599 (2)	4.216 (1)	10.773(2)
**0.5°**	9.594 (2)	4.215 (1)	10.772 (2)
**1.0°**	9.594 (2)	4.214 (1)	10.771 (2)
**2.0°**	9.594 (2)	4.214 (1)	10.772 (2)
**Literature data**			
R. Viennoise et al.[1]	9.600(1)	4.2140(5)	10.783(1)

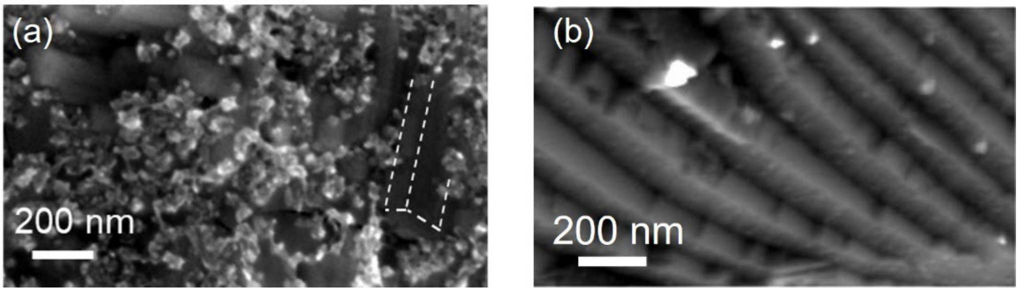

Figure S3. (a) Scanning electron microscopy (SEM) image (top view) from the combinatorial sample area with [Cu]/([Ba]+[Cu]) ≈ 0.67, i. e., a composition around the 1:2:2 stoichiometry of $BaCu_2Se_2$. The dashed lines are guides to the eye. (b) SEM image (top view) of a 2.3 µm thick $BaCu_2Se_2$ thin film with [Cu]/([Ba]+[Cu]) = 0.68 deposited on a Mo coated soda lime glass substrate.

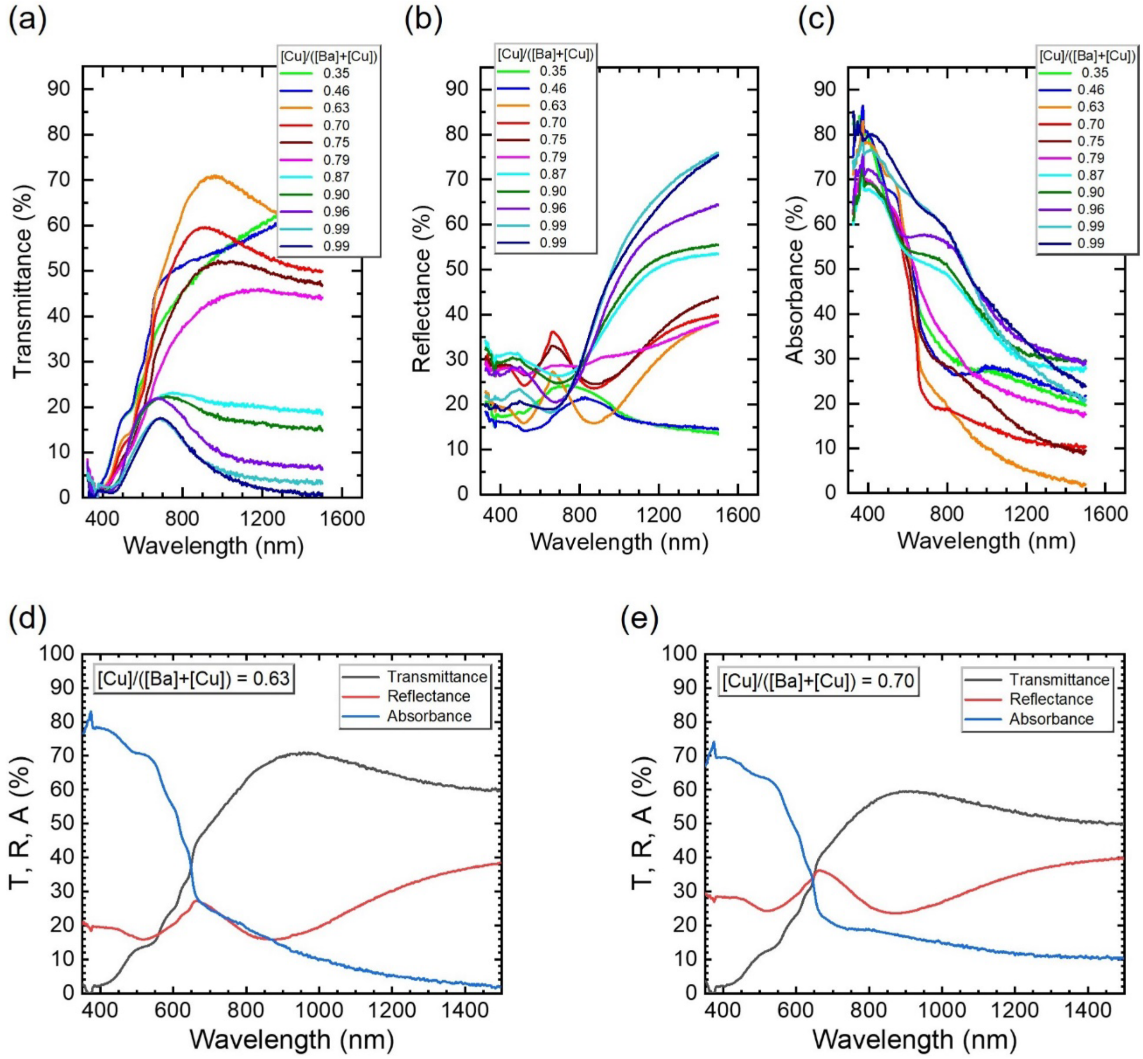

Figure S4. UV-vis-IR (a) transmittance and (b) reflectance spectra of the combinatorial Ba-Cu-Se sample as function of [Cu]/([Ba]+[Cu]) ratio between 0.35 and 0.99. (c) Absorbance spectra calculated with the measured transmittance and reflectance data. (d) Transmittance, reflectance and absorbance spectra of $BaCu_2Se_2$ with the composition around 1:2:2 stoichiometry, i.e., [Cu]/([Ba]+[Cu]) ≈ 0.67, e.g. of (d) [Cu]/([Ba]+[Cu]) = 0.63 and (e) [Cu]/([Ba]+[Cu]) = 0.70.

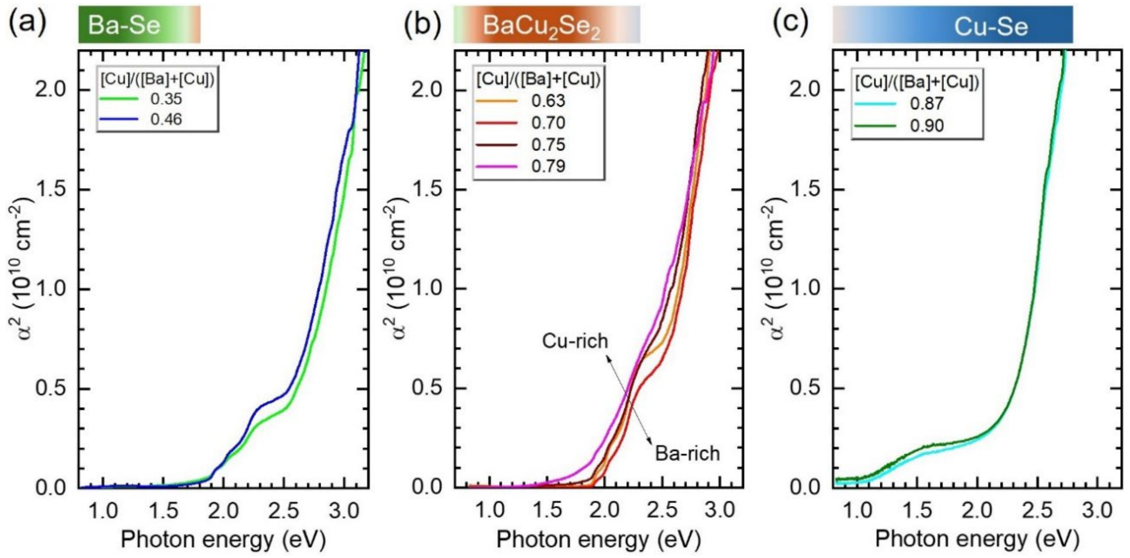

Figure S5. Squared absorption coefficient spectra of the Ba-Cu-Se combinatorial sample from different areas dominated by (a) Ba-Se phases such as BaSe and $BaSe_3$ with an amount of about 26 wt.% of α-$BaCu_2Se_2$ phase, (b) α-$BaCu_2Se_2$ with an amount of about 1 wt.% BaSe and 6 wt.% $BaSe_3$, and (c) Cu-Se phases such as CuSe and Cu_2Se as function of different [Cu]/([Ba]+[Cu]) ratios. The respective phase fractions are given according to data presented in Figure 2b of the manuscript.

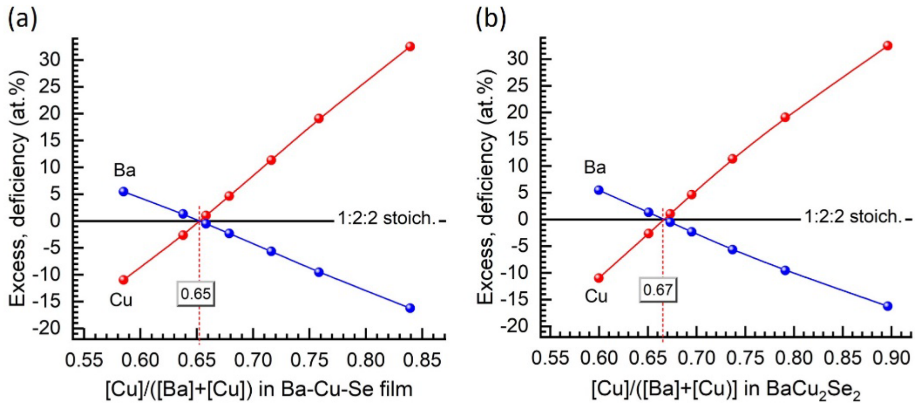

Figure S6. (a) Excess-deficiency elemental concentrations of Ba and Cu as calculated relative to the 1:2:2 stoichiometry of the $BaCu_2Se_2$ phase in Ba-Cu-Se thin films as a function of the [Cu]/([Ba]+[Cu]) atomic ratio as obtained from XRF analysis. The interception point of the Ba and Cu curves shows that the 1:2:2 stoichiometric $BaCu_2Se_2$ in Ba-Cu-Se library is achieved at a [Cu]/([Ba]+[Cu]) = 0.65. (b) Excess-deficiency elemental concentrations of Ba and Cu as calculated with respect to the 1:2:2 stoichiometry of the α-$BaCu_2Se_2$ phase after subtraction from the composition of the Ba-Cu-Se film of the Se fraction, which forms the $BaSe_3$ secondary phase. The contribution of BaSe in this compositional range is neglected since its phase content is below 1 at.%.

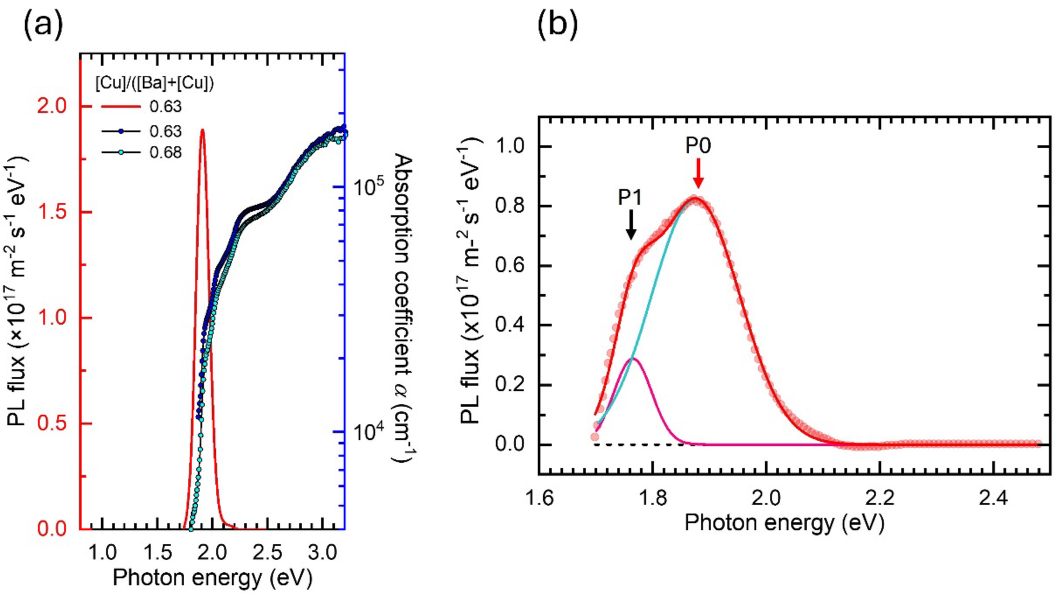

Figure S7. (a) Absolute intensity PL spectrum and absorption coefficient of $BaCu_2Se_2$ thin film for [Cu]/([Ba]+[Cu]) atomic ratios around 1:2:2 stoichiometry. (b) Analysis of the absolute intensity photoluminescence (PL) spectrum of the Ba-Cu-Se film from the area of Ba-rich compositions.

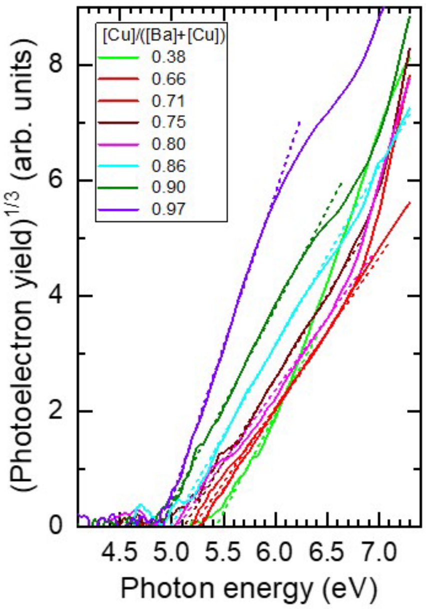

Figure S8. Photoelectron yield spectra (PYS) of the Ba-Cu-Se combinatorial sample as a function of the thin film composition. The dotted lines depict the extrapolations of the straight parts of the PYS spectra for determination of ionization energy values.

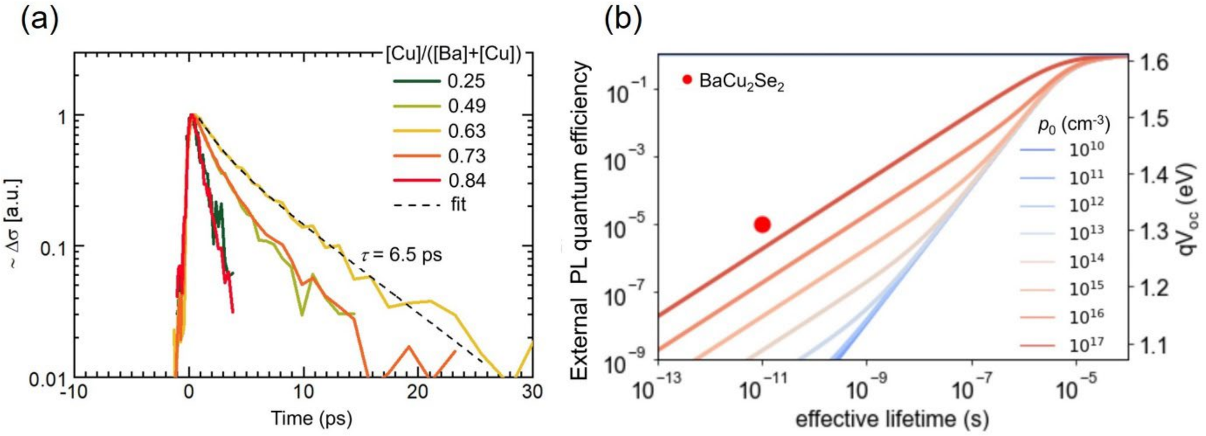

Figure S9. (a) THz photoconductivity transients measured at different positions of the sample with different [Cu]/([Ba]+[Cu]) atomic ratios. (b) Comparison of the experimental values of the external PL quantum efficiency (EQE_{PL}) and of the effective lifetime τ of charge carriers in stoichiometric $BaCu_2Se_2$ to the calculated $EQE_{PL} = f(\tau)$ dependencies and of the theoretically obtainable solar cell V_{oc} values calculated as function of τ for different equilibrium charge carrier concentrations p_0 in the absorber.

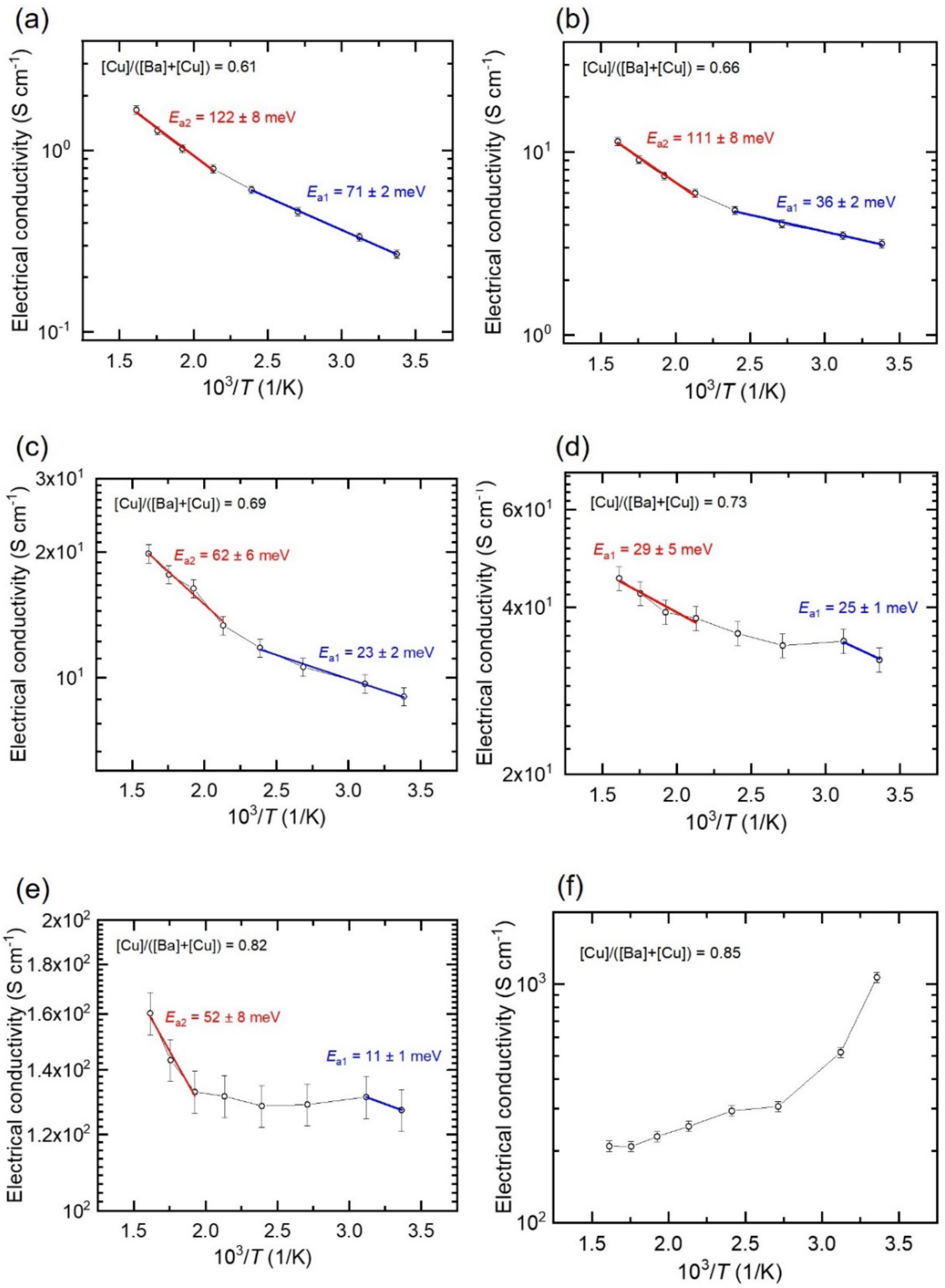

Figure S10. Arrhenius plots of electrical conductivity of Ba-Cu-Se thin films with various [Cu]/([Ba]+[Cu]) atomic ratios. Activation energies E_{a1} and E_{a2} were obtained by fitting the linear parts of the graphs in the low and high temperature regions, respectively, with the equation for conductivity $\sigma = \sigma_0 \exp\left(-E_a/kT\right)$, where σ_0 is the nominal conductivity at infinite temperature T (Kelvin) and k is the Boltzmann constant. The plots (a-e) indicate a semiconducting behavior while the plot (f) shows a metallic behavior corresponding to a degenerate semiconductor.

References

[1] R. Viennois, D. Bérardan, C. Popescu, *J. Phys. Chem. C* **2020**, *124*, 13627.

[2] T. A Grzybowski, A. L. Ruoff, *Phys. Rev. B* **1983**, 27(10), 6502.

[3] F. Hulliger, T. Siegrist, *Z. Naturforsch.* **1981**, 36b, 14.
